# Rice *Basic Helix-Loop-Helix 079* (*OsbHLH079*) Delays Leaf Senescence by Attenuating ABA Signaling

**DOI:** 10.1186/s12284-023-00673-w

**Published:** 2023-12-13

**Authors:** Suk-Hwan Kim, Jungwon Yoon, Hanna Kim, Sang-Ji Lee, Nam-Chon Paek

**Affiliations:** Department of Agriculture, Forestry and Bioresources, Plant Genomics and Breeding Institute, Research Institute of Agriculture and Life Sciences, Seoul National University, Seoul, Republic of Korea

**Keywords:** Abscisic acid signaling, Basic helix-loop-helix transcription factor, Leaf senescence, OsbHLH079, Rice, Senescence-associated transcription factor

## Abstract

Leaf senescence represents the final phase of leaf development and is characterized by a highly organized degenerative process involving the active translocation of nutrients from senescing leaves to growing tissues or storage organs. To date, a large number of senescence-associated transcription factors (sen-TFs) have been identified that regulate the initiation and progression of leaf senescence. Many of these TFs, including NAC (NAM/ATAF1/2/CUC2), WRKY, and MYB TFs, have been implicated in modulating the expression of downstream senescence-associated genes (SAGs) and chlorophyll degradation genes (CDGs) under the control of phytohormones. However, the involvement of basic helix-loop-helix (bHLH) TFs in leaf senescence has been less investigated. Here, we show that *OsbHLH079* delays both natural senescence and dark-induced senescence: Overexpression of *OsbHLH079* led to a stay-green phenotype, whereas *osbhlh079* knockout mutation displayed accelerated leaf senescence. Similar to other sen-TFs, *OsbHLH079* showed a gradual escalation in expression as leaves underwent senescence. During this process, the mRNA levels of SAGs and CDGs remained relatively low in *OsbHLH079* overexpressors, but increased sharply in *osbhlh079* mutants, suggesting that *OsbHLH079* negatively regulates the transcription of SAGs and CDGs under senescence conditions. Additionally, we found that *OsbHLH079* delays ABA-induced senescence. Subsequent RT-qPCR and dual-luciferase reporter assays revealed that OsbHLH079 downregulates the expression of ABA signaling genes, such as *OsABF2*, *OsABF4*, *OsABI5*, and *OsNAP*. Taken together, these results demonstrate that *OsbHLH079* functions in delaying leaf yellowing by attenuating the ABA responses.

**Supplementary Information:**

The online version contains supplementary material available at 10.1186/s12284-023-00673-w.

## Background

Leaves function as the primary organ for photosynthesis, converting light energy into chemical energy in the form of carbohydrate molecules (Woo et al. 2019). Leaf senescence, known as the final stage of leaf development, is a highly orchestrated degenerative process that entails the gradual breakdown of cellular components and macromolecules, including proteins, nucleic acids, and lipids (Pennell and Lam 1997; Gregersen et al. 2013; Schippers et al. 2015). The relocation of nutrients from senescing leaves towards developing tissues or storage organs is crucial for nutrient management and reproductive success (Lim et al. 2007; Masclaux-Daubresse et al. 2008). Crop yield and quality are greatly influenced by the timing of leaf senescence (Buchanan-Wollaston et al. 2005; Breeze et al. 2011; Su et al. 2017). Accelerated leaf senescence adversely impacts crop quality and yield by reducing the accumulation of photosynthetic assimilates in storage organs (Yang et al. 2016a; Mao et al. 2017). Conversely, delaying leaf senescence enables crops to prolong their photosynthetic lifespan, resulting in increased grain yield. However, it also reduces the protein level in grains due to delayed nitrogen remobilization from leaves (Zhao et al. 2015; Carmo-Silva et al. 2017; Havé et al. 2017). Hence, investigating the regulatory mechanisms of leaf senescence will offer important insights for crafting crop breeding strategies with a goal of enhancing agronomic traits.

The onset of leaf senescence depends primarily on developmental age, but is also influenced by several endogenous factors and external cues. Endogenous factors such as phytohormone signals and nutrient status play a pivotal role, while the external cues include wounding, shading, high salinity, and pathogen infection (Smart 1994; Jing et al. 2002; Moore et al. 2003; Gepstein and Glick 2013; Zakari et al. 2020). As leaves mature, a significant change in gene expression occurs (Lin and Wu 2004; van der Graaff et al. 2006). Using comprehensive global gene expression profiling methods, scientists have identified several genes that are induced during leaf senescence. These genes are referred to as senescence-associated genes (SAGs) which control the progression of leaf senescence (Buchanan-Wollaston 1997; Guo and Gan 2005; Li et al. 2012). Over the past few decades, the physiological roles of SAGs in plants have been elucidated: they are involved in diverse biological processes, including nutrient redistribution, biomolecule degradation, and phytohormone signaling (Kong et al. 2006; Li et al. 2020). For instance, *OsSAG12-2*, encodes a functional proteolytic enzyme that is induced under senescence conditions and regulates stress-induced cell death (Singh et al. 2016). *RLS1* (*Rapid Leaf Senescence 1*), which encodes an NB (nucleotide-binding site) containing protein with an ARM (armadillo) domain, is upregulated during dark-induced leaf senescence. It regulates the autophagy-like process leading to chloroplast degradation in senescent leaves (Jiao et al. 2012). Chlorophyll degradation genes (CDGs), which constitute a subset of extensively characterized SAGs, play a pivotal role in the gradual decline of chlorophyll levels in leaves during senescence. A total of seven CDGs have been identified in rice, namely *OsNYC1*, *OsNOL*, *OsHCAR*, *OsSGR*, *OsNYC3*, *OsPAO*, and *OsRCCR1*. These genes encode enzymes responsible for catalyzing sequential reactions within the chlorophyll degradation pathway and are induced when leaves enter senescence (Piao et al. 2017; Lee and Masclaux-Daubresse 2021). Loss-of-function mutations in any of these CDGs result in prominent stay-green phenotypes, primarily due to impaired chlorophyll catabolism (Kusaba et al. 2007; Park et al. 2007; Morita et al. 2009; Sato et al. 2009; Tang et al. 2011; Piao et al. 2017).

Phytohormones, including abscisic acid (ABA), ethylene (ET), jasmonic acid (JA), and salicylic acid (SA), serve as the primary regulators of leaf senescence (Lim et al. 2007; Woo et al. 2019). Among these phytohormones, ABA, classified as a sesquiterpenoid hormone, stands out as one of the extensively studied senescence-promoting phytohormones: the exogenous application of ABA noticeably accelerates leaf senescence by inducing several SAGs (Quiles et al. 1995). In recent decades, a number of ABA-related mutants have been isolated, and their senescence phenotypes have been thoroughly investigated. These studies comprehensively revealed that mutants with elevated ABA levels or enhanced ABA signaling display premature a leaf senescence phenotype, while mutants with decreased ABA levels or attenuated ABA signaling display a stay-green phenotype during senescence process (Gao et al. 2016; Huang et al. 2018; Kim et al. 2019; Piao et al. 2019). For example, a loss-of-function mutation in *OsNCED3*, an ABA biosynthetic gene in rice, results in delayed leaf senescence due to ABA deficiency, whereas *OsNCED3*-overexpressing lines with elevated ABA levels exhibit accelerated leaf senescence (Huang et al. 2018). Another example is OsWRKY5, a member of the WRKY transcription factor family in rice, which promotes leaf senescence by upregulating ABA biosynthesis genes such as *OsNCED3* and *OsNCED5* (Kim et al. 2019). In Arabidopsis, three ABA-responsive element binding factors (ABFs), including ABF2, ABF3, and ABF4, enhance ABA signaling and activate CDGs, thereby facilitating chlorophyll breakdown under ABA-induced senescence conditions (Gao et al. 2016). In contrast, OsMYB102, a rice MYB transcription factor, retards both natural leaf senescence and dark-induced senescence by attenuating ABA signaling (Piao et al. 2019).

To date, a large number of transcription factors (TFs) have been isolated in plants whose expression is activated during senescence (Guo et al. 2004). Many of these TFs, termed senescence-associated TFs (sen-TFs), operate downstream of phytohormone signaling networks and are involved in the regulation of SAGs, thereby influencing the onset and progression of leaf senescence (Bengoa Luoni et al. 2019; Woo et al. 2019). Among these TFs, NAC (NAM/ATAF1/2/CUC2), WRKY, and MYB TFs have been extensively studied, revealing their pivotal functions in orchestrating the significant reprogramming of gene expression during senescence (Miao et al. 2008; Balazadeh et al. 2011; Zhang et al. 2012; Park et al. 2018; Kim et al. 2019; Sakuraba et al. 2020). Despite these advances, the involvement of basic helix-loop-helix (bHLH) TFs in leaf senescence remains poorly understood. In this study, we shed light on this aspect by showing that OsbHLH079 retards both natural leaf senescence and dark-induced senescence. Similar to other sen-TFs, the expression of *OsbHLH079* gradually increased during leaf senescence. During leaf senescence, the SAGs and CDGs were downregulated in *OsbHLH079* overexpressors and upregulated in *osbhlh079* knockout mutants. Moreover, *OsbHLH079* was shown to delay ABA-induced senescence by attenuating ABA signaling. Collectively, our results suggest that OsbHLH079 delays leaf senescence through its role as a negative regulator in the ABA signaling pathway.

## Results

### **Expression of*OsbHLH079*is Increased during Leaf Senescence**

**Fig. 1:**
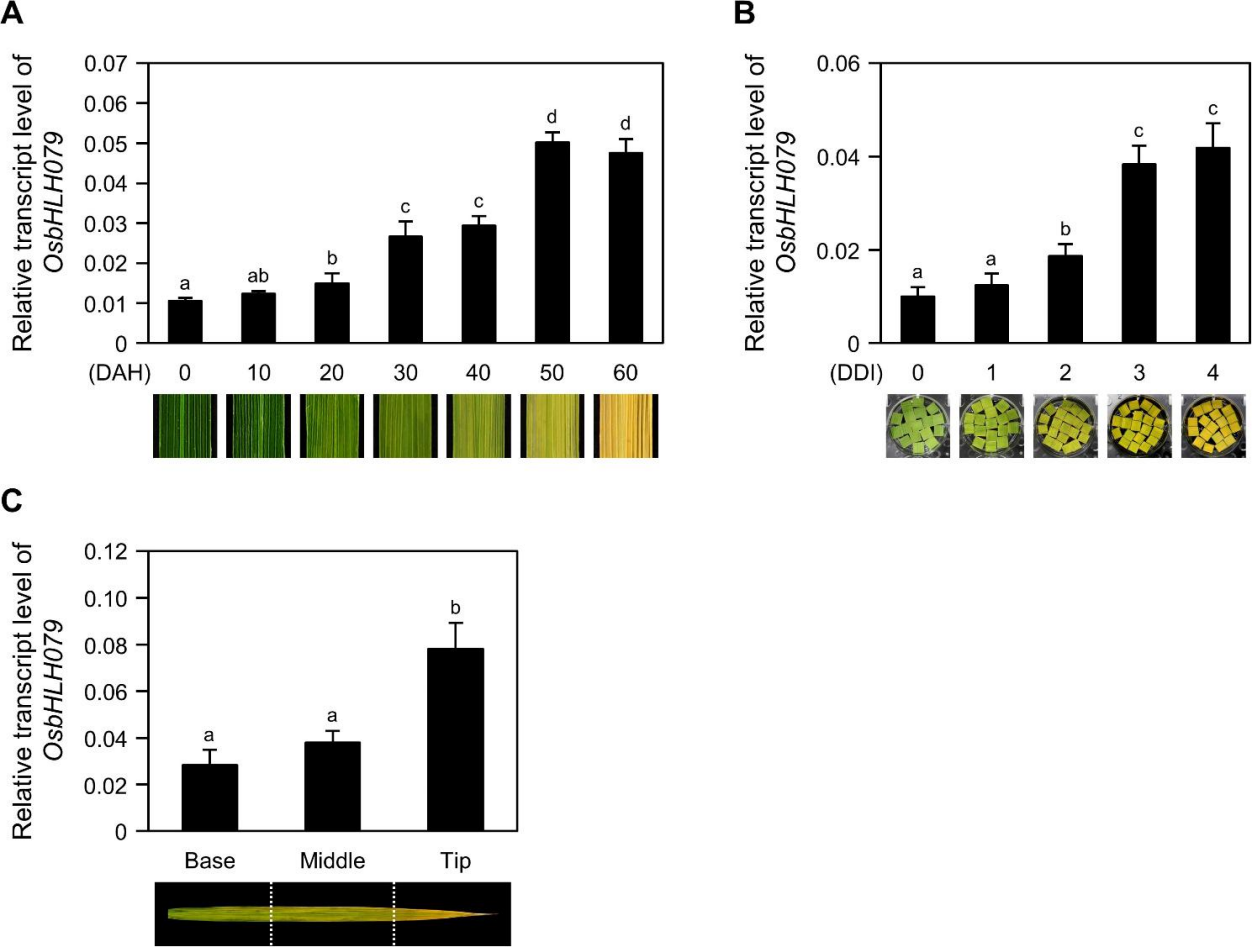
Expression profiles of *OsbHLH079* during leaf senescence. **A** Temporal expression patterns of *OsbHLH079* during natural senescence. Flag leaves were harvested at 10-day intervals from WT plants grown under natural long-day conditions in the field. The expression levels of *OsbHLH079* were determined by RT-qPCR analysis and normalized to those of *GAPDH*. Averages and standard deviations were obtained from four biological samples, each consisting of approximately five flag leaves. Representative phenotypes of flag leaves at each time point are shown as images. DAH, days after heading. **B** Relative transcript levels of *OsbHLH079* during dark-induced senescence. Leaf segments were collected from the flag leaves of WT plants at the heading stage and then floated, abaxial side up, on a 3 mM MES buffer (pH 5.8) at 30 °C in complete darkness. Leaf discs were sampled every 24 h at the indicated DDI. The expression levels of *OsbHLH079* determined by RT-qPCR analysis were normalized to those of *GAPDH*. Data represent the mean ± SD of four biological replicates (approximately 4 leaf discs per sample). Representative phenotypes of leaf discs are shown for each time point. DDI, day(s) of dark incubation. **C** Spatial expression patterns of *OsbHLH079* in senescing leaves. Each sector of naturally senescing leaves was sampled from flag leaves of WT at 50 DAH grown in the field. The mRNA levels of *OsbHLH079* were quantified by RT-qPCR analysis with *GAPDH* as an internal control. Data are presented as mean ± SD (*n* = 4). **A-C** Significantly different values are indicated by distinct letters, as determined by one-way ANOVA and Duncan’s least significant range test (*P* < 0.05). These experiments were performed twice with independent biological replicates, and similar results were obtained

OsbHLH079 (MSU locus ID, LOC_Os02g47660; RAP locus ID, Os02g0705500) is a 361 amino acid protein that belongs to the basic helix-loop-helix (bHLH) transcription factor family in rice (Li et al. 2006). The open reading frame (ORF) of *OsbHLH079* spans 1,086 bp and consists of six exons (Seo et al. 2020). To investigate the effect of senescence on the expression of *OsbHLH079*, we initially measured the total chlorophyll contents and the mRNA levels of senescence-associated genes (SAGs) in the flag leaves of the *japonica* rice cultivar ‘Dongjin’ (hereafter referred to as wild type; WT) under natural senescence or dark-induced senescence conditions. As senescence progressed, the total chlorophyll contents decreased, and the mRNA levels of SAGs increased (Additional file 1: Figures 1, 2 and 3). Next, we monitored changes in *OsbHLH079* mRNA levels in the flag leaves of WT during natural senescence. Reverse transcription and quantitative real-time PCR (RT-qPCR) analysis revealed a gradual increase in *OsbHLH079* transcript levels during natural senescence (Fig. 1A). In addition, *OsbHLH079* expression was upregulated in detached flag leaves during dark-induced senescence (DIS) (Fig. 1B). Furthermore, in naturally senescing flag leaves, *OsbHLH079* transcripts accumulated to higher levels in the yellow sector compared to the green sector (Fig. 1C). These results suggest that *OsbHLH079* is involved in the progression of leaf senescence in rice.

### ***OsbHLH079*Delays Dark-Induced Leaf Senescence**

To investigate the physiological role of *OsbHLH079* in leaf senescence, we used two independent transgenic lines overexpressing *OsbHLH079* (referred to as *osbhlh079-D* and *OsbHLH079-OE*) (See Additional file 1: Fig. S4 for details). In addition, we generated two independent knockout mutant lines (designated as *osbhlh079-1* and *osbhlh079-2*) (See Additional file 1: Fig. S5 for details). The *osbhlh079-D* line carries an activation-tagged T-DNA located 815 nucleotides upstream of the initiation codon of *OsbHLH079* (Additional file 1: Fig. S4A), and detailed genetic information on *osbhlh079-D* has been reported previously (Seo et al. 2020). The *OsbHLH079-OE* line contains *35S::OsbHLH079* constructs integrated into its genome (Additional file 1: Fig. S4B). RT-qPCR analysis revealed the significant accumulation of *OsbHLH079* transcripts in both lines compared to the WT, with the *OsbHLH079-OE* line showing even higher expression levels than the *osbhlh079-D* line (Additional file 1: Fig. S4C). The two independent *osbhlh079* mutants, *osbhlh079-1* and *osbhlh079-2*, were generated by CRISPR/Cas9-mediated mutagenesis (Miao et al. 2013), followed by the isolation of null segregants (Additional file 1: Fig. S5A, B). Chromatograms from direct sequencing of *OsbHLH079* revealed that *osbhlh079-1* and *osbhlh079-2* carry a 5-bp deletion and a 1-bp deletion, respectively, within the coding region of *OsbHLH079*, as shown in Additional file 1: Fig. S5C-F.

**Fig. 2:**
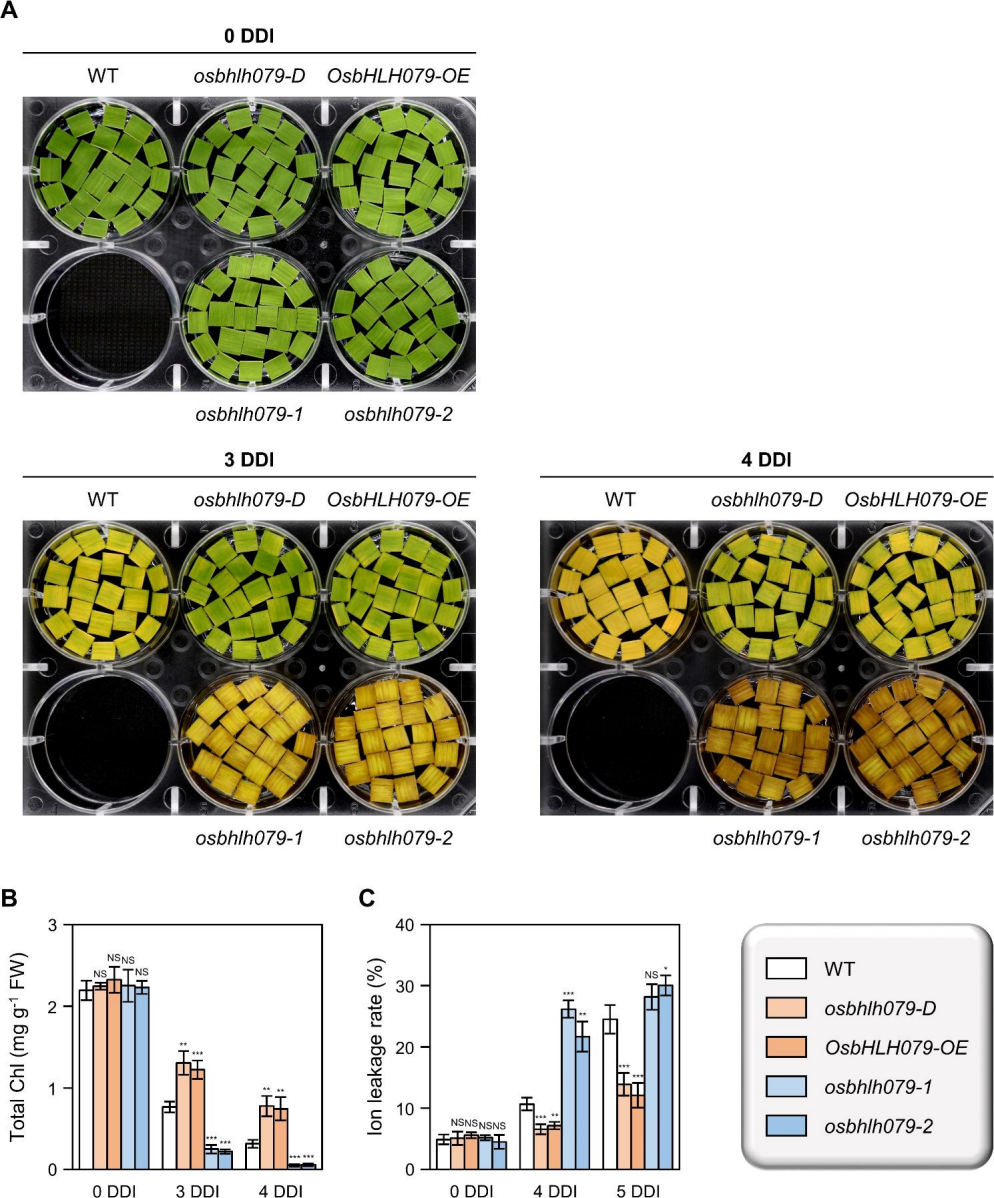
*OsbHLH079* delays leaf yellowing during dark-induced senescence. **A-C** Changes in leaf color (**A**), total chlorophyll content (**B**), and ion leakage rate (**C**) in complete darkness. Rice plants were grown under natural day-night conditions in the paddy field, and leaf discs were collected from the flag leaves of rice plants at the heading stage. The leaf discs were then incubated on a 3 mM MES buffer (pH 5.8), abaxial side up, at 30 °C in complete darkness until the indicated DDI. The data in (**B**, **C**) were obtained from four independent samples, with approximately 10 mg of leaf discs per sample in (**B**) and around five leaf discs per sample in (**C**), and are presented as mean ± SD. Statistical analysis was performed using the two-tailed Student’s *t*-test (*P < 0.05, **P < 0.01, and ***P < 0.001). These experiments were repeated twice with similar results. Chl, chlorophyll; DDI, day(s) of dark incubation; FW, fresh weight; NS, not significant

We then evaluated the phenotypes of *osbhlh079-D*, *OsbHLH079-OE*, *osbhlh079-1*, and *osbhlh079-2* grown under natural long-day conditions in the field. At the heading stage, the top four leaves of *osbhlh079-D* and *OsbHLH079-OE* displayed wider leaf angles, while those of *osbhlh079-1* and *osbhlh079-2* showed narrower leaf angles compared to the WT (Additional file 1: Fig. S6), as previously reported (Seo et al. 2020). To evaluate the effects of *OsbHLH079* on the progression of dark-induced senescence (DIS), we incubated the detached flag leaf discs from WT, *osbhlh079-D*, *OsbHLH079-OE*, *osbhlh079-1*, and *osbhlh079-2* plants on a 3 mM MES buffer (pH 5.8) at 30 °C in complete darkness. Compared to WT, the leaf discs of *OsbHLH079* overexpressors retained their green color for a longer period of time, whereas those of *osbhlh079* mutants exhibited accelerated leaf yellowing compared to WT (Fig. 2A). Consistent with the visible phenotypes, the total chlorophyll levels in the leaves of *osbhlh079-D* and *OsbHLH079-OE* remained higher, while those of *osbhlh079-1* and *osbhlh079-2* were lower than WT during DIS (Fig. 2B). Furthermore, the ion leakage rate, an indicator of membrane disintegration, was significantly lower in the leaf discs of *OsbHLH079* overexpressors and higher in those of *osbhlh079* mutants than in WT at 4 and 5 days of dark incubation (DDI) (Fig. 2C). In addition, the senescence-associated genes (SAGs) and the chlorophyll degradation genes (CDGs) were down-regulated in *OsbHLH079*-overexpressing lines and up-regulated in *osbhlh079* knock-out mutant lines under DIS conditions (Additional file 1: Fig. S7). Taken together, these results suggest a negative role of *OsbHLH079* in leaf yellowing during DIS.

### ***OsbHLH079*Retards Natural Leaf Senescence**

**Fig. 3:**
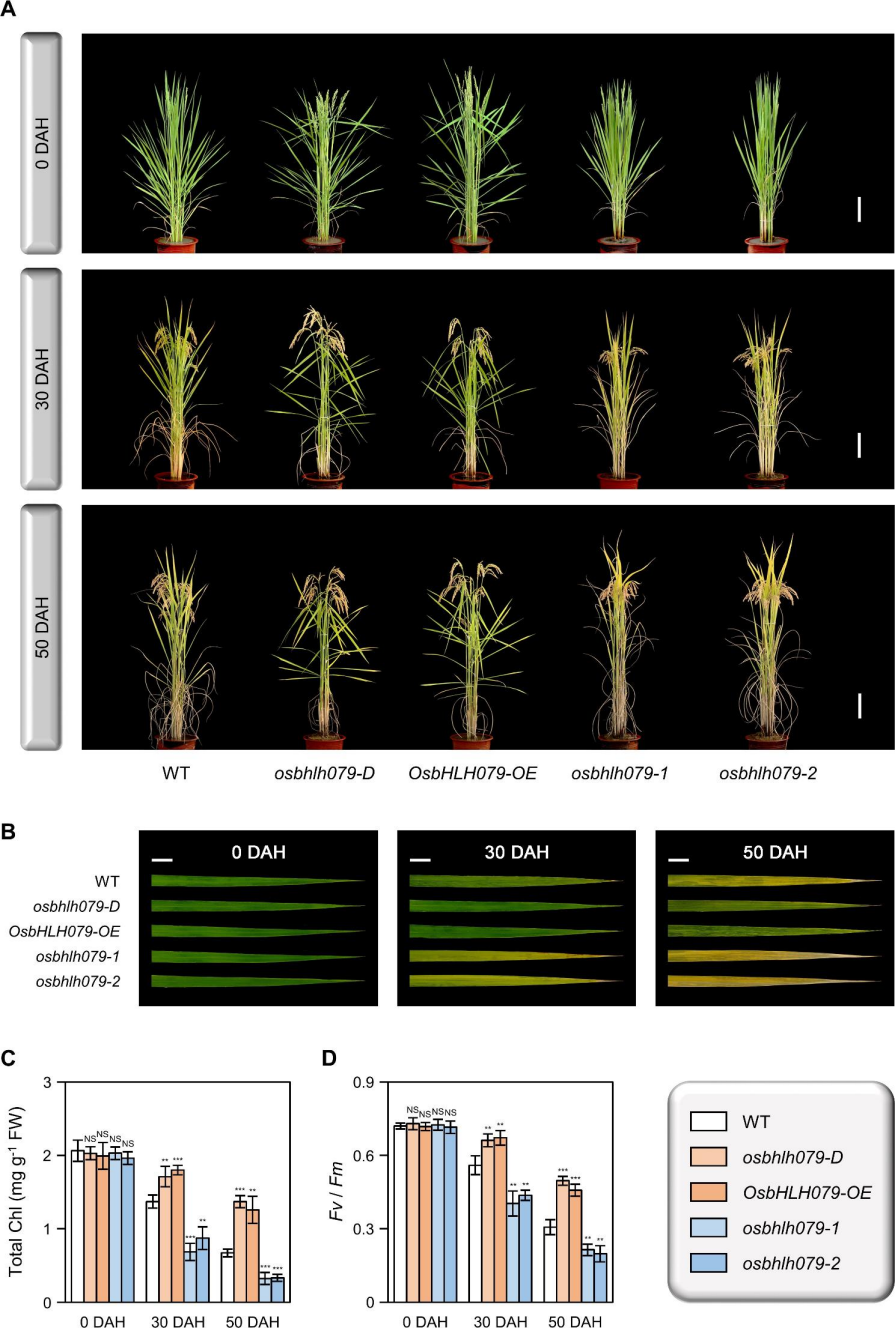
*OsbHLH079* negatively regulates natural leaf senescence in rice. **A** Phenotypes of WT, *osbhlh079-D*, *OsbHLH079-OE*, *osbhlh079-1*, and *osbhlh079-2* at 0, 30, and 50 DAH grown in the field. Scale bars = 20 cm. **B** Comparison of leaf phenotypes among WT, *osbhlh079-D*, *OsbHLH079-OE*, *osbhlh079-1*, and *osbhlh079-2* plants during natural senescence. Flag leaves from the plants in (**A**) were photographed, and the images shown are representative of three independent flag leaves. Scale bars = 2 cm. **C, D** Changes in total chlorophyll content (**C**) and *Fv*/*Fm* ratio (**D**) of flag leaves under natural senescence conditions. Means and standard deviations were obtained from four independent plants. Asterisks denote statistically significant differences compared to WT, as determined by the two-tailed Student’s *t*-test (**P < 0.01 and ***P < 0.001). These experiments were performed twice with similar results. Chl, chlorophyll; DAH, days after heading; FW, fresh weight; NS, not significant

**Fig. 4:**
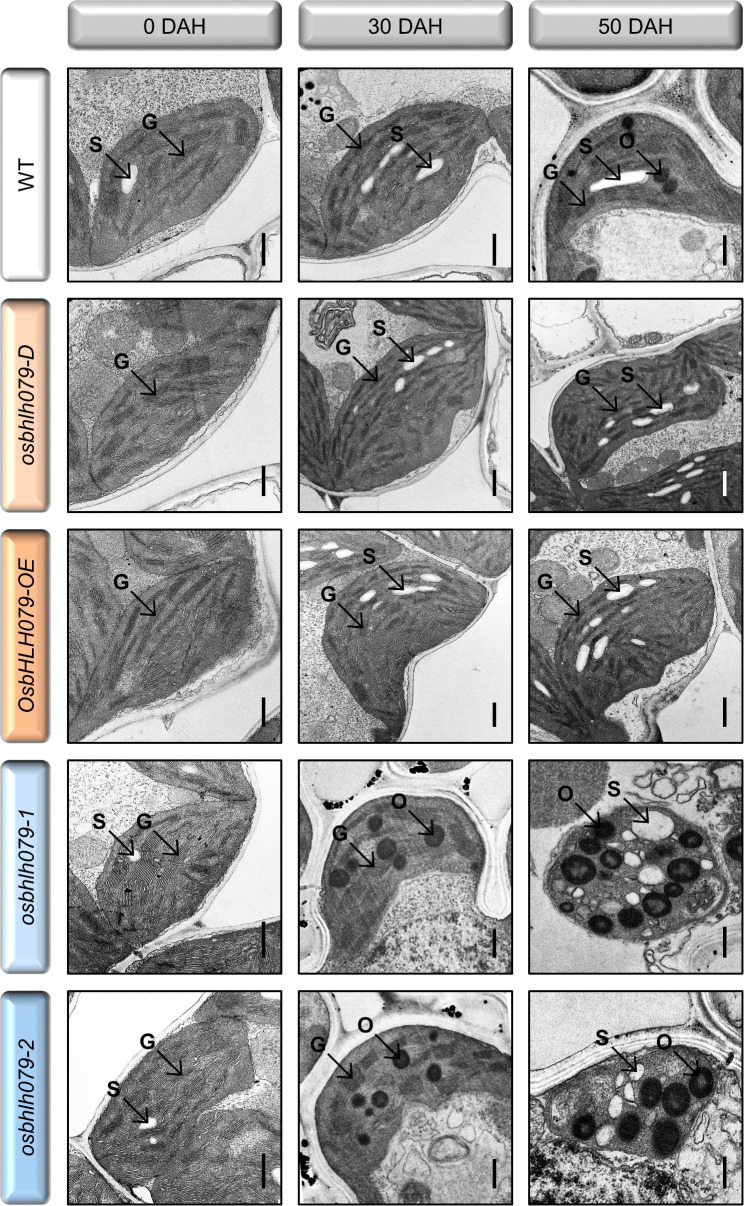
Transmission electron micrograph (TEM) of chloroplasts during natural senescence. Leaf segments, approximately 2 cm away from the leaf tip, were sampled from the flag leaves of WT, *osbhlh079-D*, *OsbHLH079-OE*, *osbhlh079-1*, and *osbhlh079-2* grown under natural field conditions and subjected to TEM analysis. Scale bars = 500 nm. The images are representative of four independent chloroplasts. DAH, days after heading; G, grana thylakoid; O, osmiophilic globule; S, starch granule

To elucidate the physiological role of *OsbHLH079* in natural leaf senescence, we monitored the progression of age-dependent leaf senescence in WT, *osbhlh079-D*, *OsbHLH079-OE*, *osbhlh079-1*, and *osbhlh079-2* grown in the natural field. At the heading stage, there were no noticeable differences in leaf color among these plants (Fig. 3A, B). However, *osbhlh079-D* and *OsbHLH079-OE* displayed a delayed leaf yellowing phenotype, whereas *osbhlh079-1* and *osbhlh079-2* showed a premature leaf yellowing phenotype during grain filling (Fig. 3A, B). Consistent with these observations, the flag leaves of *OsbHLH079* overexpressors maintained a higher concentration of total chlorophyll, whereas those of *osbhlh079* mutants showed a significant decrease in total chlorophyll content compared to the WT flag leaves during natural senescence (Fig. 3C). In addition, we measured the *Fv*/*Fm* ratio, which serves as an indicator of photosystem II efficiency, to compare the photosynthetic performance of the leaves under natural senescence conditions. The flag leaves of *OsbHLH079* overexpressors sustained higher *Fv*/*Fm* ratios than those of WT during grain filling (Fig. 3D). In contrast, the *Fv*/*Fm* ratios in the flag leaves of *osbhlh079* null mutants decreased drastically after the heading stage compared to those of WT (Fig. 3D). Next, we performed a comparative analysis of the chloroplast structure in senescing flag leaves among WT, *osbhlh079-D*, *OsbHLH079-OE*, *osbhlh079-1*, and *osbhlh079-2* using transmission electron microscopy (TEM). At the heading stage, the chloroplasts of all plants appeared intact, with highly stacked grana thylakoids (Fig. 4). However, at 30 days after heading (DAH), osmiophilic globuli, which are plastid-localized lipoprotein particles associated with senescent chloroplasts (Besagni and Kessler 2013), began to accumulate in the chloroplasts of *osbhlh079-1* and *osbhlh079-2*, whereas the chloroplasts of WT, *osbhlh079-D*, and *OsbHLH079-OE* did not show any accumulation of osmiophilic globuli (Fig. 4). At 50 DAH, the chloroplasts of *OsbHLH079* overexpressors remained intact and free of osmiophilic globuli, whereas those of WT plants accumulated osmiophilic globuli (Fig. 4). Meanwhile, the thylakoid membranes of chloroplasts in *osbhlh079-1* and *osbhlh079-2* were almost completely disintegrated, and the number and size of osmiophilic globuli were significantly increased (Fig. 4). Collectively, these results suggest that OsbHLH079 functions as a negative regulator of natural leaf senescence.

### ***OsbHLH079*Modulates the Expression of Senescence-Associated Genes during Natural Senescence**

**Fig. 5:**
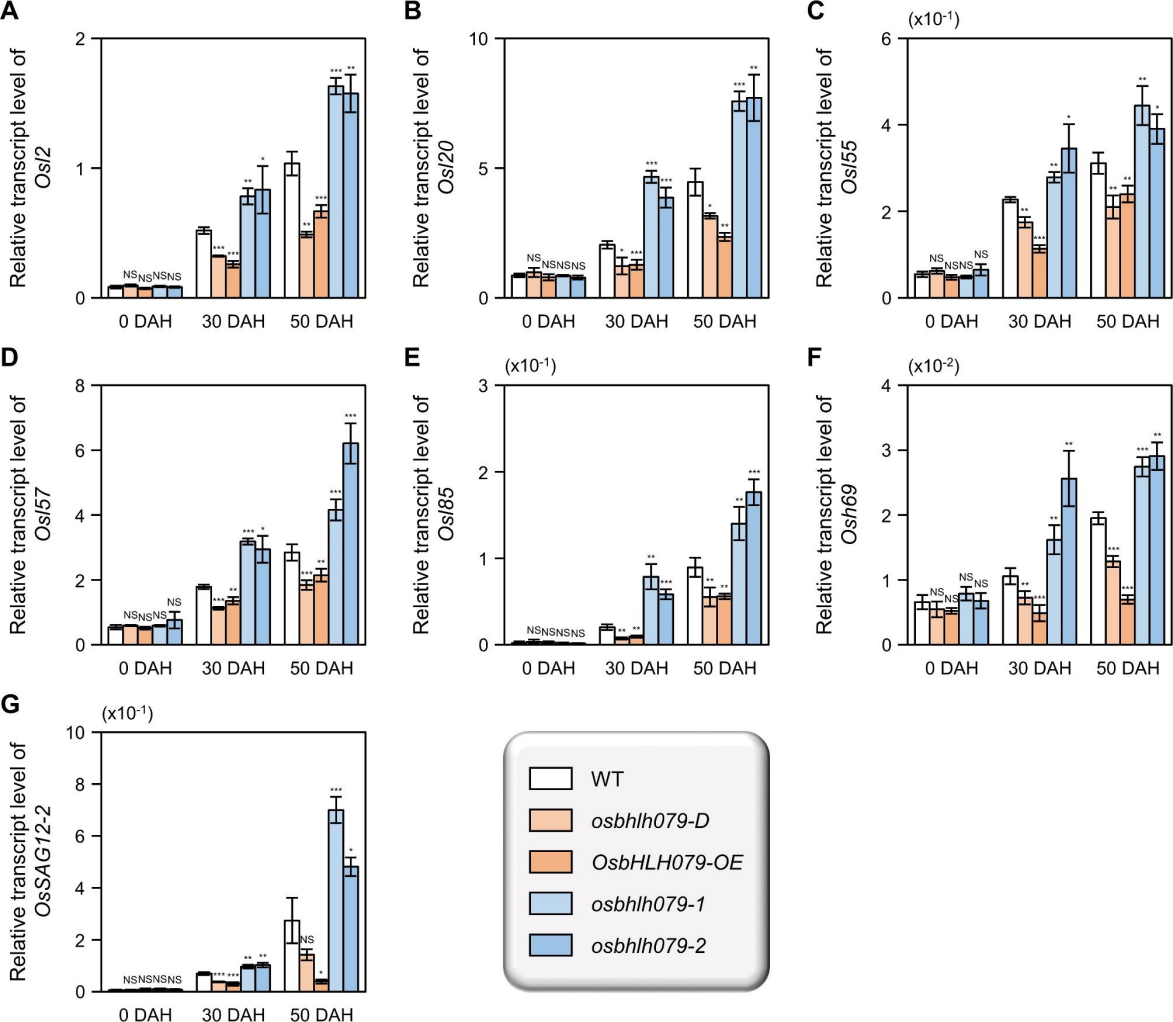
Expression profiles of senescence-associated genes in naturally senescing flag leaves. **A-G** Relative transcript levels of *Osl2* (**A**), *Osl20* (**B**), *Osl55* (**C**), *Osl57* (**D**), *Osl85* (**E**), *Osh69* (**F**), and *OsSAG12-2* (**G**) in WT, *osbhlh079-D*, *OsbHLH079-OE*, *osbhlh079-1*, and *osbhlh079-2*. The middle parts of the flag leaves were harvested from the rice plants grown under natural long-day conditions in the paddy field at the indicated DAH and were used for RT-qPCR analysis. The expression levels of each gene were normalized to that of *GAPDH*, an internal control. The values presented are the means of four biological replicates, with each replicate consisting of five independent flag leaves, and the error bars indicate the standard deviations. Significance of differences between means was analyzed by the two-tailed Student’s *t*-test (*P < 0.05, **P < 0.01, and ***P < 0.001). These analyses were performed twice independently with similar results. DAH, days after heading; NS, not significant

Senescence-associated genes (SAGs) are upregulated under senescing conditions and play a crucial role in controlling the progression of leaf senescence (Lee et al. 2001; Lee and Masclaux-Daubresse 2021). To investigate the effect of OsbHLH079 on the expression of SAGs during leaf senescence, we conducted RT-qPCR analysis to compare the mRNA levels of representative SAGs in senescing flag leaves of WT, *osbhlh079-D*, *OsbHLH079-OE*, *osbhlh079-1*, and *osbhlh079-2* grown in the natural field. Representative SAGs included *Osl2* (aminotransferase), *Osl20* (E1-α subunit of branched-chain α-keto dehydrogenase), *Osl55* (biotinylated subunit of β-methylcrotonyl-CoA carboxylase), *Osl57* (3-ketoacyl-CoA thiolase), *Osl85* (isocitrate lyase), *Osh69* (seed imbibition protein), and *OsSAG12-2* (proteolytic enzyme) (Lee et al. 2001; Singh et al. 2016) (see Additional file 2: Table S1). At the heading stage, there were no detectable differences in the expression levels of any of the genes among WT, *osbhlh079-D*, *OsbHLH079-OE*, *osbhlh079-1*, and *osbhlh079-2* (Fig. 5). However, during grain filling, the SAGs were downregulated in *OsbHLH079*-overexpressing lines and upregulated in *osbhlh079* loss-of-function mutants (Fig. 5). These results indicate that OsbHLH079 retards the progression of leaf senescence by negatively regulating the transcription of SAGs under senescent conditions.

### ***OsbHLH079*Downregulates Chlorophyll Degradation Genes during Leaf Senescence**

**Fig. 6:**
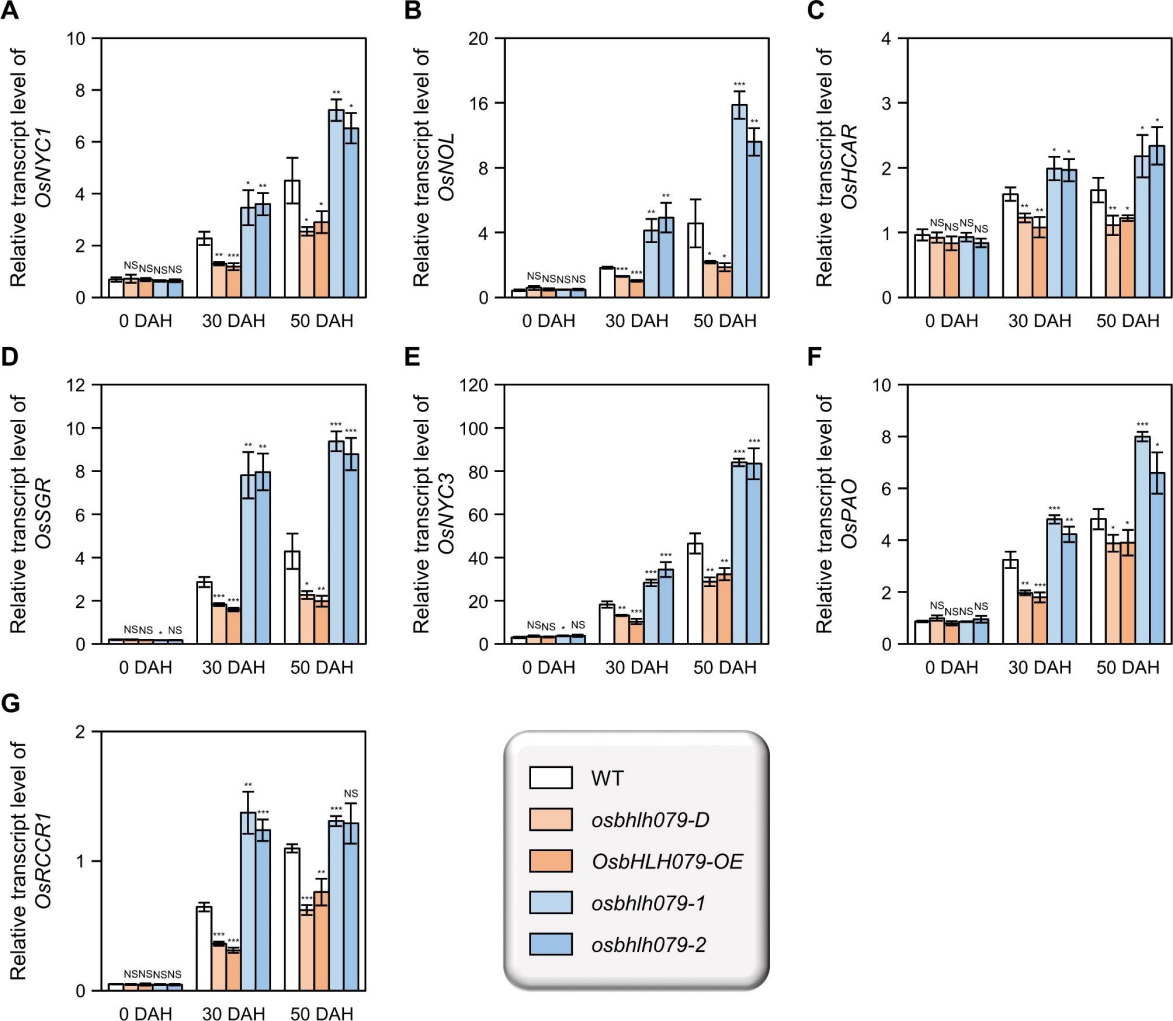
Expression analysis of chlorophyll degradation genes during natural senescence of flag leaves. **A-G** Relative mRNA levels of *OsNYC1* (**A**), *OsNOL* (**B**), *OsHCAR* (**C**), *OsSGR* (**D**), *OsNYC3* (**E**), *OsPAO* (**F**), and *OsRCCR1* (**G**) were compared among WT, *osbhlh079-D*, *OsbHLH079-OE*, *osbhlh079-1*, and *osbhlh079-2*. Total RNA samples from Fig. 5 were subjected to RT-qPCR analysis, using *GAPDH* as a reference for normalization. Data are presented as mean ± SD of four independent biological samples, each with approximately five flag leaves per sample. Asterisks on the bars indicate statistically significant differences compared to WT transcript levels (Student’s *t*-test; *P < 0.05, **P < 0.01, and ***P < 0.001). These experiments were performed twice with similar results. DAH, days after heading; NS, not significant

Chlorophyll degradation is a crucial process in leaf senescence, leading to the gradual loss of green pigments (Pružinská et al. 2005; Gan and Hörtensteiner 2013). Chlorophyll degradation genes (CDGs) encode enzymes that facilitate sequential reactions in the chlorophyll catabolic pathway, and CDGs are known to be induced under senescence conditions (Piao et al. 2017; Lee and Masclaux-Daubresse 2021). To investigate the role of OsbHLH079 in chlorophyll degradation, we compared the transcript levels of CDGs, including *OsNYC1* (chlorophyll *b* reductase; Kusaba et al. 2007), *OsNOL* (chlorophyll *b* reductase; Sato et al. 2009), *OsHCAR* (7-hydroxy methyl chlorophyll *a* reductase; Piao et al. 2017), *OsSGR* (Mg^2+^ dechelatase; Park et al. 2007), *OsNYC3* (pheophytinase; Morita et al. 2009), *OsPAO* (pheophorbide *a* oxygenase; Tang et al. 2011), and *OsRCCR1* (red chlorophyll catabolite reductase; Tang et al. 2011), in flag leaves of WT, *osbhlh079-D*, *OsbHLH079-OE*, *osbhlh079-1*, and *osbhlh079-2* grown under natural long-day conditions. Consistent with previous reports (Piao et al. 2017; Lee and Masclaux-Daubresse 2021), their transcript abundances gradually increased in WT as senescence progressed. At the heading stage, the expression levels of the CDGs in *osbhlh079-D*, *OsbHLH079-OE*, *osbhlh079-1*, and *osbhlh079-2* were similar to those in WT (Fig. 6). However, the mRNA levels of the CDGs in senescing flag leaves were lower in *OsbHLH079* overexpressors and higher in *osbhlh079* knockout mutants compared to those in WT (Fig. 6). These results suggest that OsbHLH079 functions as a repressor of CDG expression during natural senescence, thereby inhibiting the progression of leaf yellowing.

### ***OsbHLH079*Regulates ABA-induced Leaf Senescence in Rice**

**Fig. 7:**
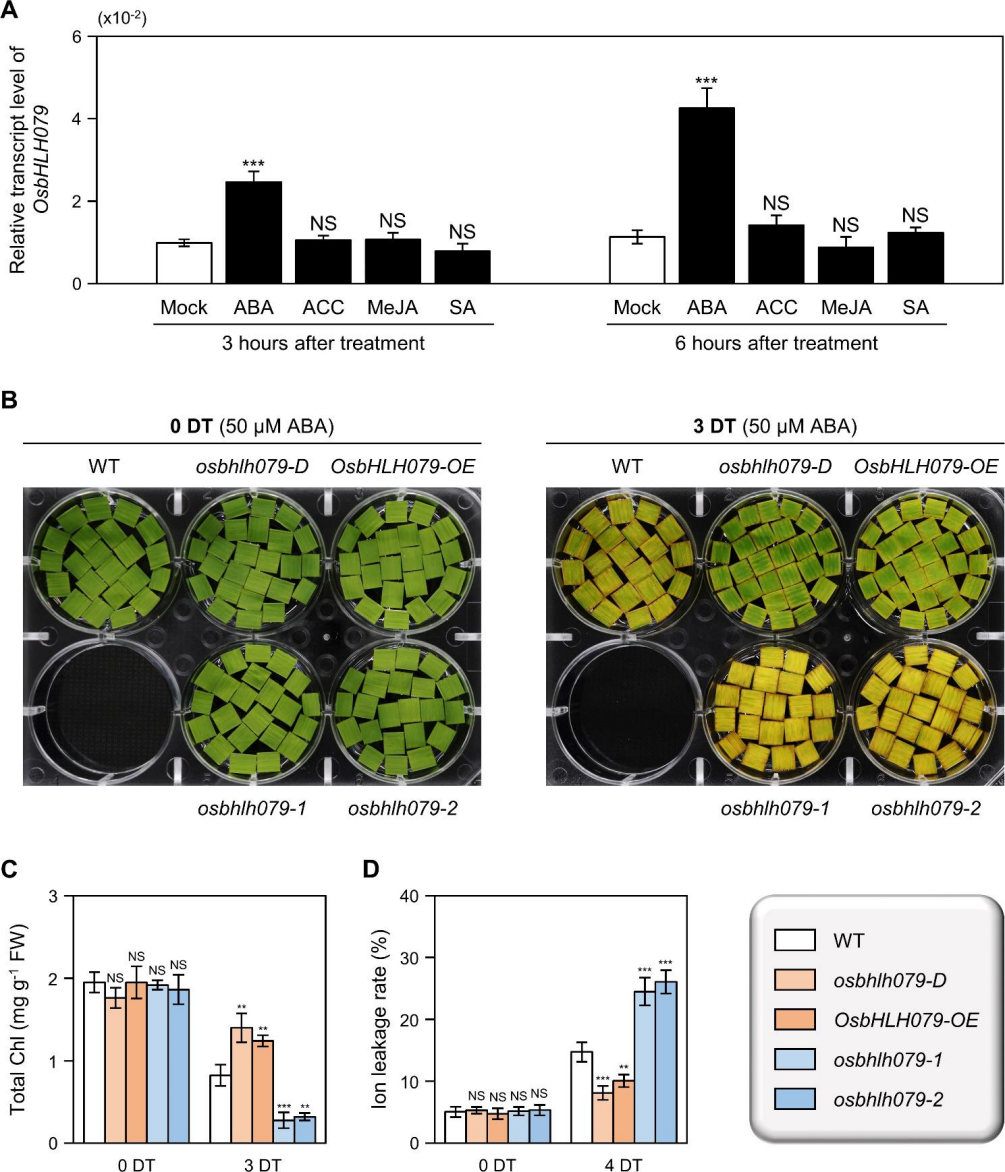
*OsbHLH079* delays ABA-induced leaf senescence in rice. **A** Expression of *OsbHLH079* in response to senescence-promoting phytohormones. WT seedlings were grown on half-strength Murashige and Skoog (MS) solid medium (pH 5.7) under constant light conditions at 30 °C for 10 days. Seedlings were then transferred to half-strength MS liquid medium supplemented with 100 µM ABA, 10 mM ACC, 100 µM MeJA, or 100 µM SA. Leaves were harvested at 3 and 6 h after treatments, and their total RNAs were used for RT-qPCR analysis, with expression levels of *OsbHLH079* normalized to those of *GAPDH*. Means and standard deviations were obtained from four biological replicates consisting of approximately three leaves per sample. Statistically significant differences compared to the mock-treated samples are marked by asterisks, as determined by the two-tailed Student’s *t*-test (***P < 0.001). ABA, abscisic acid; ACC, 1-aminocyclopropane-1-carboxylic acid; MeJA, methyl jasmonate; NS, not significant; SA, salicylic acid. **B-D** Leaf color (**B**), total chlorophyll content (**C**), and ion leakage rate (**D**) of WT, *osbhlh079-D*, *OsbHLH079-OE*, *osbhlh079-1*, and *osbhlh079-2* under ABA-induced senescence conditions. Flag leaf discs were collected at the heading stage from the rice plants grown under natural field conditions. The leaf discs were then treated with a 3 mM MES buffer (pH 5.8) containing 50 µM ABA for the indicated time periods under continuous light conditions at 30 °C. Data presented in (**C**, **D**) represent the average ± SD of four biological replicates [around 10 mg of leaf discs per replicate in (**C**) and approximately five leaf discs per replicate in (**D**)]. Asterisks indicate significantly different values according to Student’s *t*-test (**P < 0.01 and ***P < 0.001). ABA, abscisic acid; Chl, chlorophyll; DT, day(s) of treatment; FW, fresh weight; NS, not significant. These experiments were performed twice with similar results

Various phytohormones, such as abscisic acid (ABA), ethylene (ET), jasmonic acid (JA), and salicylic acid (SA), play important roles in the progression of leaf senescence (Jan et al. 2019; Guo et al. 2021; Huang et al. 2022). To investigate the effects of these phytohormones on *OsbHLH079* expression, we measured the mRNA levels of *OsbHLH079* in the leaves of 10-day-old WT seedlings treated with ABA, 1-aminocyclopropane-1-carboxylic acid (ACC, the immediate precursor of ET; Ververidis and John 1991), methyl jasmonate (MeJA, the methylated derivative of JA; Peng and Zhang 2017), or SA. RT-qPCR analysis revealed that *OsbHLH079* transcription was exclusively induced in response to ABA: *OsbHLH079* expression increased to approximately 2.5-fold and 3.9-fold after 3 and 6 h of ABA treatment, respectively (Fig. 7A). Next, we monitored the progression of leaf yellowing in detached flag leaves of WT, *osbhlh079-D*, *OsbHLH079-OE*, *osbhlh079-1*, and *osbhlh079-2* during treatment with ABA, ACC, MeJA, or SA. Before the application of phytohormones, leaf colors were nearly uniform in each plant line (Fig. 7B and Additional file 1: Fig. S8). After 3 days of treatments, we observed that *OsbHLH079* overexpressors and *osbhlh079* knockout mutants showed hyposensitivity and hypersensitivity, respectively, to ABA-induced leaf senescence: the leaves of *osbhlh079-D* and *OsbHLH079-OE* retained their green colors for a longer period of time compared to those of WT, while the leaf discs of *osbhlh079-1* and *osbhlh079-2* exhibited an early leaf yellowing phenotype (Fig. 7B). In support of these observations, the *osbhlh079-D* and *OsbHLH079-OE* maintained high levels of total chlorophyll, whereas the total chlorophyll contents in *osbhlh079-1* and *osbhlh079-2* sharply decreased during ABA-induced leaf senescence (Fig. 7C). Moreover, the ion leakage rates of leaf discs from *OsbHLH079* overexpressors remained at a low level, while those of *osbhlh079* knockout mutants dramatically increased under ABA-mediated senescence conditions (Fig. 7D). In addition, the transcript levels of SAGs and CDGs were lower in *OsbHLH079*-overexpressing lines and higher in *osbhlh079* knock-out mutant lines compared to those in WT at 3 days of ABA treatment (Additional file 1: Fig. S9). However, during ACC-, MeJA-, or SA-mediated senescence, leaf colors, total chlorophyll levels, and ion leakage rates showed almost no discernible differences among WT, *osbhlh079-D*, *OsbHLH079-OE*, *osbhlh079-1*, and *osbhlh079-2* (Additional file 1: Figs. S8 and S10). Taken together, these results indicate that OsbHLH079 plays a crucial role in delaying ABA-induced leaf senescence in rice.

### ***OsbHLH079*Suppresses the Expression of ABA Signaling Genes under Senescing Conditions**

**Fig. 8:**
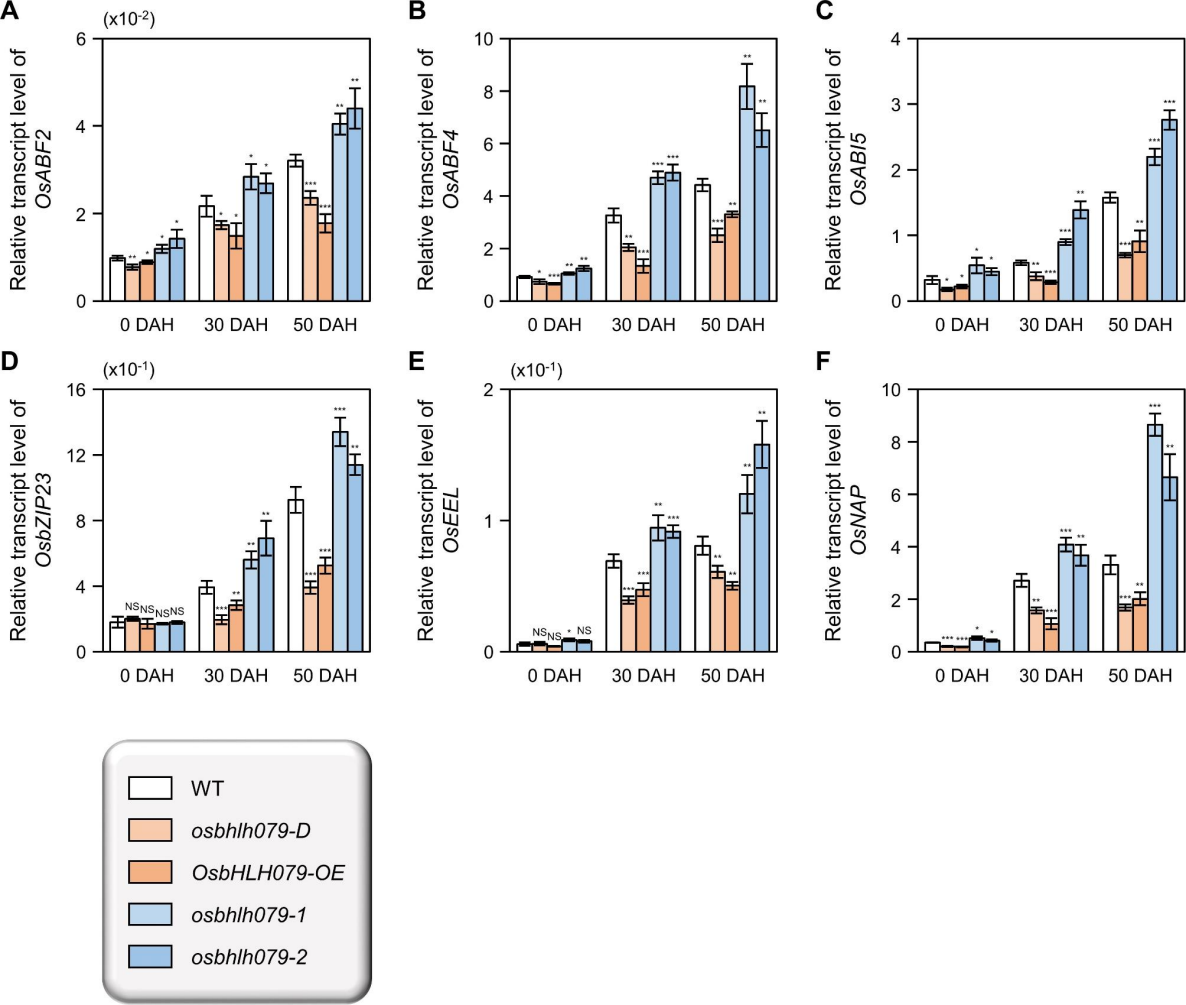
Transcriptional analysis of genes involved in ABA signaling during natural leaf senescence in rice. **A-F** Relative transcript abundances of *OsABF2* (**A**), *OsABF4* (**B**), *OsABI5* (**C**), *OsbZIP23* (**D**), *OsEEL* (**E**), and *OsNAP* (**F**) were measured in flag leaves of WT, *osbhlh079-D*, *OsbHLH079-OE*, *osbhlh079-1*, and *osbhlh079-2*. Total RNAs isolated from the samples shown in Fig. 5 were subjected to RT-qPCR analysis. The mRNA levels of *GAPDH*, a reference gene, were used to normalize. The values shown in the graphs are averages of four independent samples (around 5 flag leaves per sample), and the error bars represent standard deviations. Differences between the means were statistically analyzed using two-tailed Student’s *t*-test (*P < 0.05, **P < 0.01, and ***P < 0.001). These analyses were repeated twice with similar results. DAH, days after heading; NS, not significant

**Fig. 9:**
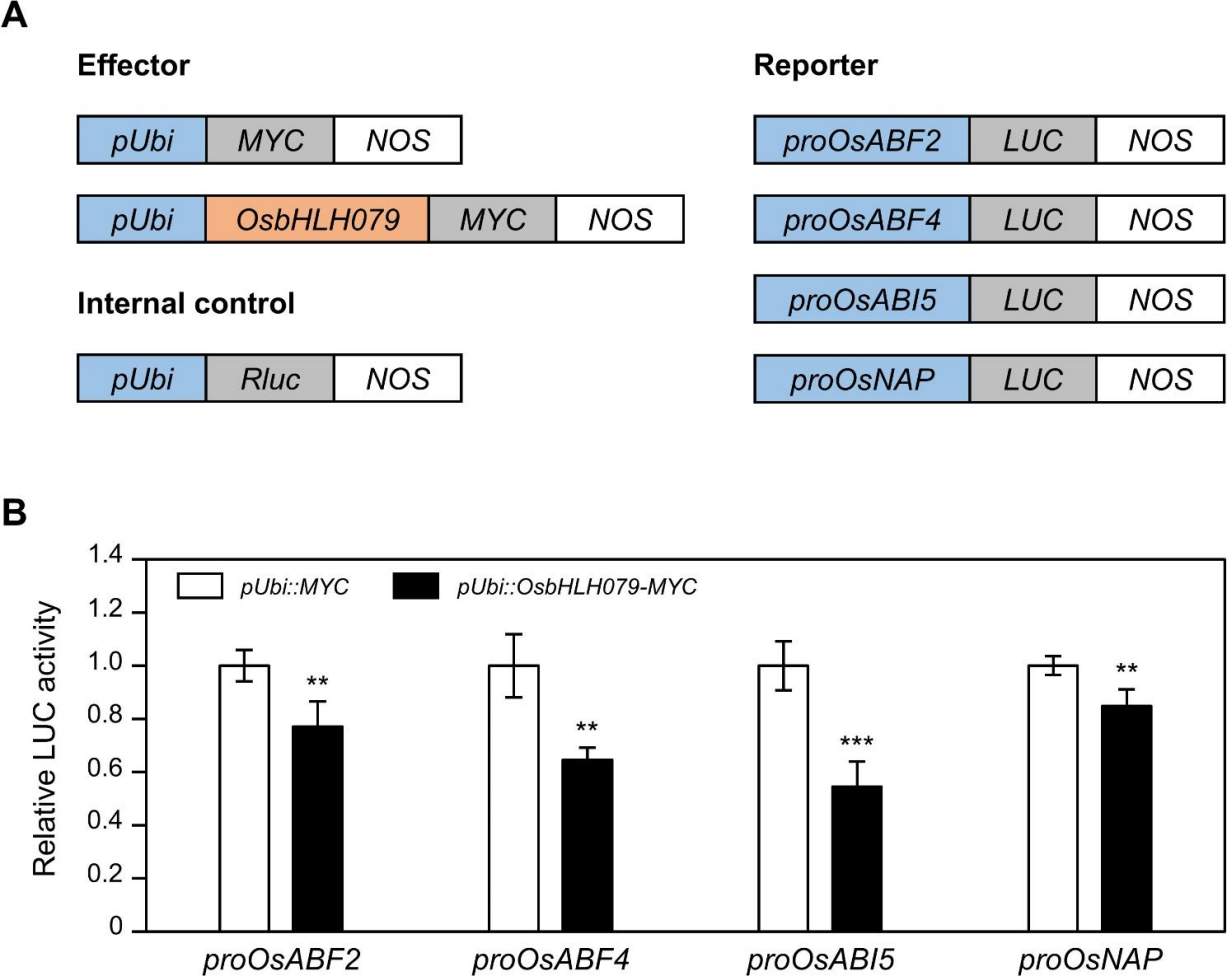
OsbHLH079 represses the transcription of *OsABF2*, *OsABF4*, *OsABI5*, and *OsNAP*. **A** Description of the constructs used in the dual-luciferase reporter assay, including the effectors, reporters, and internal control. *LUC* was fused to *proOsABF2* (-1,553 to -1), *proOsABF4* (-1,709 to + 7), *proOsABI5* (-1,971 to + 58), or *proOsNAP* (-1,542 to + 84). LUC, luciferase; NOS, NOS terminator; pUbi, promoter of *Ubiquitin*; Rluc, renilla luciferase. **B** The repression of *proOsABF2*, *proOsABF4*, *proOsABI5*, and *proOsNAP* by OsbHLH079-MYC expression in the dual-luciferase reporter assay. MYC was used as a negative control. The LUC activity of each sample was represented relative to that of the negative control, set as 1. Averages and standard deviations were obtained from five independent samples. Significant differences between means were determined using a two-tailed Student’s *t*-test (**P < 0.01, and ***P < 0.001). LUC, luciferase; pUbi, promoter of *Ubiquitin*

Since OsbHLH079 delays ABA-induced leaf senescence (Fig. 7B-D), we hypothesized that OsbHLH079 might be related to ABA metabolism and/or signaling. To investigate the role of OsbHLH079 in ABA metabolism, we compared the endogenous ABA levels in flag leaves among WT, *osbhlh079-D*, *OsbHLH079-OE*, *osbhlh079-1*, and *osbhlh079-2*. However, no significant differences in endogenous ABA levels were found among the plant lines (Additional file 1: Fig. S11). We then focused on the relationship between OsbHLH079 and ABA signaling genes. Specifically, we analyzed the expression patterns of ABA signaling genes, including *OsABF2*, *OsABF4*, *OsABI5*, *OsbZIP23*, *OsEEL*, and *OsNAP* (Xiang et al. 2008; Lu et al. 2009; Hossain et al. 2010a, b; Liang et al. 2014; Yang et al. 2019), in senescing flag leaves of WT, *osbhlh079-D*, *OsbHLH079-OE*, *osbhlh079-1*, and *osbhlh079-2*. At the heading stage, the mRNA levels of *OsABF2*, *OsABF4*, *OsABI5*, and *OsNAP* were downregulated in *OsbHLH079* overexpressors and upregulated in *osbhlh079* knock-out mutants (Fig. 8). During grain filling, the transcript abundances of all the investigated genes remained at a relatively low level in flag leaves of *OsbHLH079* overexpressors and sharply increased in the flag leaves of *osbhlh079-1* and *osbhlh079-2* knockout mutants compared to WT (Fig. 8): their expression showed an increase in WT as senescence progressed, consistent with previous studies (Hossain et al. 2010a; Liang et al. 2014; Kang et al. 2019; Piao et al. 2019; Sakuraba et al. 2020) (Fig. 8). To further confirm whether OsbHLH079 inhibits the transcription of ABA signaling genes, we conducted a dual-luciferase reporter assay. For the reporter constructs, the promoter regions of *OsABF2* (-1,553 to -1), *OsABF4* (-1,709 to + 7), *OsABI5* (-1,971 to + 58), or *OsNAP* (-1,542 to + 84) were fused with the *LUC* reporter gene (Fig. 9A). The LUC activities of the protoplasts transformed with *proOsABF2::LUC*, *proOsABF4::LUC*, *proOsABI5::LUC*, and *proOsNAP::LUC* constructs were significantly reduced when each of them was co-transfected with the *pUbi::OsbHLH079-MYC* effector plasmid (Fig. 9B). Taken together, these results suggest that OsbHLH079 desensitizes ABA signaling by down-regulating the expression of ABA signaling genes, thereby delaying leaf senescence in rice.

## Discussion

### OsbHLH079 Negatively Regulates ABA-Induced Leaf Senescence

Basic helix-loop-helix proteins are a large family of transcription factors that are widely distributed in fungi, plants, and animals (Carretero-Paulet et al. 2010; Pires and Dolan 2010). Within the plant kingdom, bHLH TFs play crucial roles in numerous biological processes, including responses to biotic and abiotic stresses, reproductive organ development, embryo growth, and hormonal signaling (Friedrichsen et al. 2002; Gremski et al. 2007; Kondou et al. 2008; Ariyarathne and Wone 2022). For example, overexpression of *AhHLH112* in peanuts enhances drought tolerance by increasing endogenous ABA levels (Li et al. 2021). In Arabidopsis, a mutation in *DYSFUNCTIONAL TAPETUM1* (*DYT1*), which encodes a bHLH TF, leads to male sterility by downregulating several tapetum-preferential genes (Zhang et al. 2006). Recently, bHLH TFs have been reported to modulate leaf senescence in Arabidopsis: *MYC2*, *MYC3*, and *MYC4* promote jasmonic acid-mediated senescence (Qi et al. 2015), whereas *ATBS1-INTERACTING FACTOR 2* (*AIF2*) delays brassinosteroid (BR)-mediated leaf senescence (Kim et al. 2020). However, the role of bHLH TFs in rice leaf senescence has not been extensively investigated. This study shows that *OsbHLH079* acts to delay both natural and dark-induced leaf senescence. Overexpression of *OsbHLH079* delays leaf senescence, while *osbhlh079* knockout mutants exhibit early leaf senescence (Figs. 2 and 3). In addition, OsbHLH079 represses the expression of ABA signaling genes, including *OsABF2*, *OsABF4*, *OsABI5*, and *OsNAP* (Figs. 8 and 9). This suggests that *OsbHLH079* suppresses ABA signaling during leaf senescence. Consistent with these data, *OsbHLH079* was shown to delay ABA-induced leaf senescence (Fig. 7B-D). Therefore, we propose that *OsbHLH079* functions to inhibit leaf senescence by desensitizing ABA signaling.

### OsbHLH079 Functions as a Negative Regulator of Leaf Senescence

Senescence-associated transcription factors (Sen-TFs), whose expressions are elevated during leaf senescence, are categorized into senescence-promoting and senescence-inhibiting types (Lee and Masclaux-Daubresse 2021). In this study, we observed that *OsbHLH079*-overexpressing lines exhibited a stay-green phenotype, whereas *osbhlh079* knock-out mutant lines displayed premature leaf yellowing (Figs. 2 and 3), indicating that OsbHLH079 functions as a senescence-inhibiting sen-TFs. Recently, several sen-TFs have been found to play roles in delaying leaf senescence. For example, ONAC106, a senescence-associated NAC TF in rice, negatively regulates leaf senescence (Sakuraba et al. 2015). Similarly, OsMYB102, a MYB-type TF in rice, retards both natural senescence and dark-induced senescence: the expression of *OsMYB102* was increased during leaf senescence, and *OsMYB102*-overexpressing plants exhibited delayed leaf senescence phenotypes (Piao et al. 2019). These types of TFs are believed to function antagonistically to senescence-promoting sen-TFs in regulating leaf senescence (Sakuraba et al. 2015; Cao et al. 2023). For instance, MYC2, MYC3, and MYC4 redundantly promote JA-mediated leaf senescence by directly up-regulating *SAG29*; meanwhile, bHLH03, bHLH13, bHLH14, and bHLH17 attenuate MYC2/MYC3/MYC4-mediated senescence by directly repressing *SAG29* in Arabidopsis (Qi et al. 2015). Therefore, we propose that OsbHLH079 antagonizes senescence-promoting sen-TFs, ensuring that leaf senescence initiates at the proper time for survival under fluctuating environmental conditions.

### OsbHLH079 Retards Leaf Senescence by Dampening ABA Signaling, Not by Reducing ABA Accumulation

To date, several sen-TFs that govern ABA-mediated leaf senescence have been identified (Park et al. 2018; Piao et al. 2019; Sakuraba et al. 2020; Xie et al. 2022). In many cases, these sen-TFs regulate both ABA metabolism and ABA signaling. For example, OsMYB102, a MYB-type TF in rice, retards leaf senescence by inhibiting ABA accumulation and attenuating ABA signaling under senescing conditions (Piao et al. 2019). In addition, OsWRKY53, a WRKY TF in rice, increases endogenous ABA levels to promote leaf senescence; the expression levels of several ABA signaling genes were also higher in *OsWRKY53*-overexpressing plants compared to the WT (Xie et al. 2022). Some sen-TFs, however, have been found to regulate leaf senescence by modulating ABA signaling, rather than by influencing ABA metabolism. For instance, ONAC054, a NAC TF in rice, promotes ABA-induced leaf senescence by activating ABA signaling genes, including *OsABF4* and *OsABI5*; conversely, the endogenous ABA contents in *onac054* mutants were nearly identical to those in the WT (Sakuraba et al. 2020). Similarly, OsRL3, a MYB-related TF in rice, up-regulates ABA signaling genes, such as *OsRK1*, *OsRAB16C*, and *OsRAB16D*, to accelerate dark-induced senescence; meanwhile, the expression levels of ABA biosynthetic genes were unchanged in *osrl3* mutants compared to the WT (Park et al. 2018). These findings indicate that sen-TFs controlling ABA-induced leaf senescence do not necessarily modulate ABA metabolism under senescing conditions. In this study, we found that OsbHLH079 delays ABA-mediated leaf senescence (Fig. 7B). Subsequent RT-qPCR and dual-luciferase reporter assays revealed that OsbHLH079 suppresses the transcriptions of downstream ABA signaling genes, including *OsABF2*, *OsABF4*, *OsABI5*, and *OsNAP* (Figs. 8 and 9). Interestingly, the endogenous ABA levels in flag leaves of *osbhlh079-D*, *OsbHLH079-OE*, *osbhlh079-1*, and *osbhlh079-2* plants were almost the same as the WT (Additional file 1: Fig. S11). Therefore, we propose that OsbHLH079 governs ABA-mediated leaf senescence by dampening ABA signaling, rather than by reducing ABA contents, as observed in the cases of ONAC054 and OsRL3.

### ***OsbHLH079*-mediated Attenuation of ABA Signaling during Leaf Senescence may be Attributed to the Antagonistic Interaction between BR and ABA**

BR, a class of plant-specific polyhydroxylated steroid hormones, play an important role in the regulation of various physiological processes, including cell elongation, cell wall regeneration, fruit ripening, pollen development, and root growth (Hacham et al. 2011; Vogler et al. 2014; Peres et al. 2019). In recent decades, there has been increasing evidence that BR also affects leaf senescence. Mutants with increased BR levels or enhanced BR signaling tend to undergo accelerated leaf senescence, whereas mutants with reduced BR levels or suppressed BR signaling show a delay in leaf senescence (Li et al. 1996; Li and Chory 1997; Yin et al. 2002; Husar et al. 2011). In addition, application of exogenous epibrassinolide, an active form of BR, leads to premature leaf senescence in a dose-dependent manner (Sağlam-Çağ 2007). These studies highlight the importance of BRs as essential phytohormones that promote senescence in plants.

In our previous study (Seo et al. 2020), we found that *OsbHLH079* regulates leaf angle and kernel length in a BR-dependent manner. *OsbHLH079* overexpressors exhibited exaggerated leaf angles and elongated grains, whereas transgenic lines with suppressed *OsbHLH079* expression displayed upright leaves and shorter grains, similar to mutants with altered BR signaling. BR signaling genes were upregulated in the *OsbHLH079* overexpressors and downregulated in the *OsbHLH079*-targeted RNAi lines. Consistent with these data, overexpression of *OsbHLH079* resulted in increased responsiveness to epibrassinolide in a BR-induced lamina joint (LJ) tilt assay, highlighting the role of *OsbHLH079* in amplifying BR signaling and consequently increasing leaf angle and kernel length. In this study, we demonstrated the critical role of *OsbHLH079* in leaf senescence. Interestingly, it appeared that BR do not serve as the primary phytohormone regulating *OsbHLH079*-mediated leaf senescence, since *OsbHLH079*, a positive regulator of the BR pathway, actually induced a delay in leaf senescence (Figs. 2 and 3). Instead, *OsbHLH079* was found to attenuate ABA signaling, thereby delaying the process of leaf senescence (Figs. 7 and 8).

To date, several molecular and physiological studies have provided substantial evidence that ABA and BR function in an antagonistic manner to control a variety of biological processes, such as seed germination, stomatal movement, and root growth (Xue et al. 2009; Hu and Yu 2014; Clouse 2016). For instance, *BIN2*-overexpressing lines, which are defective in BR signaling, were hypersensitive to ABA during seed germination in Arabidopsis (Hu and Yu 2014). Similarly, the BR-deficient Arabidopsis mutant *det2* displayed enhanced responsiveness to ABA-induced suppression of root elongation (Xue et al. 2009). More recently, a molecular link between ABA and BR signaling pathways was uncovered in Arabidopsis: BZR1, a master TF in BR signaling, directly binds to G-box *cis*-elements within the promoter of *ABA INSENSITIVE 5* (*ABI5*) and inhibits its expression (Yang et al. 2016b). Interestingly, our study demonstrated that OsbHLH079 stimulated the transcription of *OsBZR1* (Seo et al. 2020), the closest counterpart of Arabidopsis *BZR1* in rice, whereas downregulating the expression of *OsABI5*, a functional homolog of Arabidopsis *ABI5* (Fig. 8C). Based on these findings, we speculate that the increased expression of *OsBZR1* driven by OsbHLH079 may contribute to *OsbHLH079*-mediated dampening of ABA signaling, although we cannot exclude the possibility that *OsbHLH079* influences ABA signaling through a mechanism independent of BR. Taken together, our results provide insights into the interplay between ABA and BR signaling pathways in the context of leaf senescence.

### ***OsbHLH079*-mediated Suppression of ABA Signaling Alters Expression of SAGs and CDGs**

Numerous ABA signaling genes, including *OsABF2*, *OsABF4*, *OsABI5*, *OsbZIP23*, *OsEEL*, and *OsNAP*, have been found in the rice genome, and their involvement in abiotic stress response has been extensively elucidated (Xiang et al. 2008; Lu et al. 2009; Hossain et al. 2010a, b; Chen et al. 2014; Yang et al. 2019). For example, the transgenic rice overexpressing *OsABF4* exhibited enhanced sensitivity to ABA, resulting in increased drought tolerance (Lu et al. 2009). In contrast, T-DNA insertional knockout mutants of *OsABF2* or *OsABI5* displayed susceptibility to drought and salinity due to the impaired ABA response (Hossain et al. 2010a, b). Interestingly, recent investigations have unveiled a number of sen-TFs capable of influencing the expression of these ABA signaling genes, thereby controlling leaf senescence (Kang et al. 2019; Piao et al. 2019; Sakuraba et al. 2020). For instance, *ONAC054* acts as a positive regulator of leaf senescence by driving the transcription of *OsABF4* and *OsABI5* (Sakuraba et al. 2020). In addition, OsMYB102 delays both natural senescence and DIS by repressing *OsNAP* and *OsABF4* (Piao et al. 2019). In parallel, the function of *OsbHLH079* was confirmed in inhibiting the expression of various ABA signaling genes, including *OsABF2*, *OsABF4*, *OsABI5*, and *OsNAP*, ultimately contributing to the delay of leaf senescence (Figs. 8 and 9). Apparently, all these sen-TFs play a central role in the modulation of ABA-mediated leaf senescence.

To date, there is increasing evidence that ABA signaling genes themselves are capable of orchestrating the expression of SAGs and/or CDGs (Chen et al. 2014; Piao et al. 2019; Sakuraba et al. 2020). For example, OsABF4 directly induces the expression of *OsNYC1* and *OsSGR* (Piao et al. 2019). In addition, OsABI5 was also shown to directly transactivate *OsNYC1* and *OsSGR* (Sakuraba et al. 2020). Furthermore, chromatin immunoprecipitation (ChIP) assays revealed that OsNAP binds to the promoters of several SAGs and CDGs, such as *Osl57*, *Osh69*, *OsNYC1*, *OsSGR*, *OsNYC3*, and *OsRCCR1* (Chen et al. 2014). Therefore, we propose that OsbHLH079-mediated alterations in the expression of ABA signaling genes could consequently induce the downregulation of SAGs and CDGs during the process of leaf senescence, although we cannot exclude the alternative possibility that OsbHLH079 may directly inhibit the expression of SAGs and CDGs through various feed-forward regulatory loops.

## Conclusions

In this study, we show that OsbHLH079, a bHLH TF in rice, is involved in the process of leaf senescence. Similar to other sen-TFs, *OsbHLH079* exhibited a progressive increase in its expression during leaf senescence. Overexpression of *OsbHLH079* delayed leaf senescence, whereas the loss-of-function mutation in *OsbHLH079* induced premature leaf senescence. RT-qPCR analysis revealed that OsbHLH079 negatively regulates the expression of SAGs and CDGs during leaf senescence. Furthermore, we found that OsbHLH079 retards ABA-induced leaf senescence and substantially suppresses the expression of key ABA signaling genes, including *OsABF2*, *OsABF4*, *OsABI5*, and *OsNAP*. Hence, we propose that OsbHLH079 acts to attenuate ABA signaling, ultimately leading to a delay in the progression of leaf senescence in rice.

## Materials and Methods

### Plant Materials and Growth Conditions

The activation-tagged T-DNA insertion line of *OsbHLH079*, designated as *osbhlh079-D* (PFG_3A-01275), was obtained from the Salk Institute Genomics Analysis Laboratory (http://signal.salk.edu/cgi-bin/RiceGE) (Jeon et al. 2000; Jeong et al. 2002). The genetic information of *osbhlh079-D* has been described previously (Seo et al. 2020). For this study, the rice (*Oryza sativa*) plants, including *osbhlh079-D*, *OsbHLH079-OE*, *osbhlh079-1*, *osbhlh079-2*, and their parental *japonica* cultivar ‘Dongjin’ (referred to as wild type; WT), were grown under natural long-day conditions (approximately 14 h of light per day) in a paddy field located in Suwon, South Korea (37ºN latitude). The seeds were sown on seedbeds and grown in a greenhouse for one month before transplanting in the paddy field. Rice cultivation followed common agricultural practices adapted to Korean rice varieties.

### Vector Construction and Rice Transformation

To generate the *OsbHLH079-OE* transgenic plant, the full-length coding region of *OsbHLH079* was amplified by polymerase chain reaction (PCR) using cDNA obtained from leaves of the WT as a template and gene-specific primers (see Additional file 2: Table S2). The PCR product was subcloned into the pCR^TM^8/GW/TOPO® entry vector (Invitrogen, Carlsbad, CA, USA) and transferred into the pMDC32 Gateway-compatible binary destination vector (Curtis and Grossniklaus 2003) through an LR recombination reaction using the Gateway™ LR Clonase™ II Enzyme Mix (Invitrogen). To generate the *osbhlh079-1* and *osbhlh079-2* mutants, a specific 20-nt spacer sequence, GACGTTTCACGACACCGGAA, was designed using the CRISPRdirect software (https://crispr.dbcls.jp/) (Naito et al. 2015) and subcloned into a guide RNA expression cassette in the pOs-sgRNA entry vector (Miao et al. 2013). The resulting cassette was then transferred into the pH-Ubi-cas9-7 destination vector containing a Cas9 expression cassette (Miao et al. 2013) via the LR reaction.

The *Agrobacterium tumefaciens* strain LBA4404 (Ooms et al. 1982) was transformed with the resulting constructs, respectively, using the freeze-thaw method (Höfgen and Willmitzer 1988), and the calli generated from mature WT seed embryos were subjected to the *Agrobacterium*-mediated transformation of rice according to the previously described protocol (Jeon et al. 2000). The transformed calli were selected on 2N6 medium containing 50 mg L^− 1^ hygromycin (Duchefa Biochemie, Haarlem, The Netherlands) and regenerated into transgenic rice plants, including *OsbHLH079-OE*, *osbhlh079-1*, and *osbhlh079-2*, respectively.

### Reverse Transcription and Quantitative Real-Time PCR (RT-qPCR) Analysis

Total RNAs were isolated from rice leaves using the MG Total RNA Extraction Kit (MGmed, Seoul, Republic of Korea) according to the manufacturer’s instructions. The extracted RNAs were reverse transcribed to generate first-strand cDNAs using the Oligo(dT)_15_ Primer (Promega, Madison, WI, USA) and M-MLV Reverse Transcriptase (Promega). The resulting product mixtures were diluted four-fold with distilled water. Quantitative real-time PCR (qPCR) was performed using the GoTaq® qPCR Master Mix (Promega) and a LightCycler® 480 system (Roche, Basel, Switzerland). The qPCR reaction mix, with a final volume of 20 µl, was prepared by combining 2 µl first-strand cDNA mixture, 0.4 µl 10 µM forward primer, 0.4 µl 10 µM reverse primer, 10 µl GoTaq® qPCR Master Mix, and 7.2 µl nuclease-free water. Gene-specific primers are listed in Additional file 2: Table S2. The qPCR conditions consisted of an initial denaturation step at 95 °C for 2 min followed by 50 cycles of 95 °C for 15 s and 60 °C for 1 min. Data obtained by qPCR were analyzed using the 2^−ΔΔ*C*T^ method (Livak and Schmittgen 2001) with *OsGAPDH* as the reference gene for normalization (Jain et al. 2006).

### Dark Treatments

For the dark treatment, leaf discs were harvested from the flag leaves of rice plants at the heading stage, which were grown under natural day-night conditions in the paddy field. The leaf discs were then carefully placed abaxial side up on a 3 mM MES buffer (pH 5.8) and incubated for the indicated periods in complete darkness in an artificial growth chamber maintained at 30 °C.

### Phytohormone Treatments

To examine the expression level of *OsbHLH079* under different phytohormone treatments, WT seeds were sterilized in 70% (v/v) aqueous ethanol for 10 min and in 2% (w/v) sodium hypochlorite for 20 min. The sterilized seeds were then rinsed three times with sterile water and germinated on half strength Murashige and Skoog (MS) solid medium (pH 5.7) under continuous light conditions (100 µmol m^− 2^ s^− 1^) at 30 °C in an artificial growth chamber. After 10 days of growth, the seedlings were transferred to a half-strength MS liquid medium containing 100 µM ABA (Duchefa Biochemie), 10 mM ACC (Sigma-Aldrich, Saint Louis, MO, USA), 100 µM MeJA (Sigma-Aldrich), or 100 µM SA (Sigma-Aldrich). Seedlings in a half-strength MS liquid medium without additional phytohormones were used as a mock. Leaves were harvested at 3 and 6 h after treatment, and their total RNA was isolated for further analysis.

To evaluate the senescence phenotype in response to the senescence-associated phytohormone treatments, leaf discs were collected from flag leaves of WT, *osbhlh079-D*, *OsbHLH079-OE*, *osbhlh079-1*, and *osbhlh079-2* plants at the heading stage, grown under natural long-day conditions in the rice field. Leaf discs were then floated, abaxial side up, on a 3 mM MES buffer (pH 5.8) containing 50 µM ABA (Duchefa Biochemie), 20 mM ACC (Sigma-Aldrich), 50 µM MeJA (Sigma-Aldrich), or 100 µM SA (Sigma-Aldrich), followed by incubation for the indicated periods under continuous light conditions (100 µmol m^− 2^ s^− 1^) at 30 °C in an artificial growth chamber. Leaf discs that were incubated on a 3 mM MES buffer (pH 5.8) without any phytohormones were used as a control.

### Total Chlorophyll Quantification

To determine the total chlorophyll content, approximately 10 mg of flag leaves were weighed into a 2 ml microcentrifuge tube and homogenized in liquid nitrogen using a TissueLyser II (Qiagen, Hilden, Germany). The ground leaves were then dissolved in 500 µl of 80% (v/v) ice-cold acetone, followed by centrifugation at 12,000 rpm for 15 min at 10 °C. The absorbance of the supernatant was measured at wavelengths of 663 nm and 647 nm using a UV/VIS spectrophotometer (PowerWave X, BioTek, Winooski, VT, USA). Finally, total chlorophyll concentrations were calculated according to Porra et al. (1989).

### Measurement of Ion Leakage Rates

Ion leakage rates were determined as previously described by Fan et al. (1997) with minor adjustments. Five rice leaf discs, each approximately 1 cm^2^ in size, were placed in 6 ml of 0.4 M mannitol (Duchefa Biochemie) with gentle rotation for 3 h at room temperature. The initial conductivity of the solution was then measured with a conductivity meter (CON 6, LaMotte, Maryland, USA). After incubation at 90 °C for 30 min, the total conductivity of the solution was measured with the same conductivity meter (CON 6, LaMotte). Finally, the ion leakage rate was calculated as the percentage of the initial conductivity divided by the total conductivity.

### **Determination of*Fv*/*Fm*Ratios**

The *Fv*/*Fm* ratios, which represent the maximum quantum efficiency, were measured using a chlorophyll fluorometer (OS30p_+_, Opti-Sciences Inc., New Hampshire, USA). To ensure the complete oxidation of QA, a bound plastoquinone, the middle part of each flag leaf of rice plants grown in the paddy field was dark adapted for 15 min before measuring the *Fv*/*Fm* ratio. Each plant was subjected to three experimental replicates.

### Transmission Electron Microscopy (TEM)

Flag leaves were collected from rice plants grown in the paddy field and prepared for TEM analysis. Sample preparation followed the conventional method described by Inada et al. (1998) using a microwave tissue processor (PELCO BioWave® Pro+, Ted Pella, Redding, CA, USA) with microwave irradiation, as previously reported (Mowery and Bauchan 2018), with some modifications. Briefly, samples were vacuum-infiltrated in modified Karnovsky’s fixative (2% [w/v] paraformaldehyde and 2% [w/v] glutaraldehyde in 50 mM sodium cacodylate buffer, pH 7.2) for 60 min, followed by overnight incubation at 4 °C in complete darkness. The specimens were then washed three times with 50 mM sodium cacodylate buffer, pH 7.2, for 10 min each at 4 °C before postfixation with 1% (w/v) osmium tetroxide in 50 mM sodium cacodylate buffer (pH 7.2) using a microwave tissue processor. The postfixed specimens were rinsed twice with distilled water at room temperature and stained *en bloc* with 0.5% (w/v) uranyl acetate using the microwave tissue processor. To dehydrate the samples, the microwave-assisted dehydration method was used with a graded series of increasing concentrations of ethanol (one change each in 30% [v/v], 50% [v/v], 70% [v/v], and 90% [v/v] aqueous ethanol, followed by three changes in 100% [v/v] ethanol). After dehydration, the specimens were treated twice with propylene oxide under microwave irradiation and infiltrated gradually with increasing concentrations of Spurr’s resin (Spurr 1969) in propylene oxide: one change each in 20% (v/v), 40% (v/v), 60% (v/v), and 80% (v/v) Spurr’s resin, followed by two changes in 100% (v/v) Spurr’s resin, all performed using the microwave tissue processor. The specimens were then embedded in 100% (v/v) Spurr’s resin, polymerized at 70 °C for 24 h in an oven, and sectioned into 70 nm sections using an ultramicrotome (EM UC7, Leica Microsystems, Wetzlar, Germany) equipped with a Diatome diamond knife. The resulting sections were mounted on Formvar-coated copper grids (EMS, Hatfield, PA, USA), and stained with 2% (w/v) uranyl acetate and Reynolds’ lead citrate (Reynolds 1963) for 7 min each at room temperature. Finally, the chloroplast structure in each sample was observed under a transmission electron microscope (Talos L120C, FEI, Czech Republic) operating at 120 kV. Additional information on the detailed microwave procedures used for sample preparation can be found in Additional file 2: Table S3.

### Determination of ABA Contents

Flag leaves at the heading stage were collected from WT, *osbhlh079-D*, *OsbHLH079-OE*, *osbhlh079-1*, and *osbhlh079-2* grown in the natural rice field. The flag leaves were pulverized in liquid nitrogen using a mortar and pestle, and then subjected to lyophilization for 48 h in a freeze dryer (Bondiro, ilShin® Lab Co. Ltd., Yang-Ju, Republic of Korea). Approximately 150 mg of the freeze-dried sample was carefully weighed and placed in a 5 ml snap-cap centrifuge tube. The sample was dissolved in 1.5 ml of 80% (v/v) methanol containing 1 mM butylated hydroxytoluene (Sigma-Aldrich) as an antioxidant using an ultrasonic bath (Powersonic 420, Hwashin Tech Co. Ltd., Gwangju, Republic of Korea) at 0 °C for 15 min, followed by overnight rotation at 4 °C in complete darkness. After centrifugation at 4,000 g for 15 min at 4 °C, the supernatant was transferred to a 1.5 ml light-proof centrifuge tube and analyzed by enzyme-linked immunosorbent assay (ELISA) using an ABA ELISA kit (MyBioSource, San Diego, CA, USA) according to the manufacturer’s instructions. Absorbance at 450 nm was measured using a UV/VIS spectrophotometer (PowerWave X, BioTek), and ABA levels were determined using a standard curve.

### Dual-luciferase Reporter Assay

To construct the reporter plasmids containing the *LUC* reporter gene under the control of various promoters, promoter fragments of *OsABF2* (-1,553 to -1), *OsABF4* (-1,709 to + 7), *OsABI5* (-1,971 to + 58), or *OsNAP* (-1,542 to + 84) were cloned, respectively, into the pJD301 vector (Luehrsen et al. 1992). For the effector plasmids, the cDNA of *OsbHLH079* was cloned upstream of a sequence encoding six copies of a MYC epitope tag in the pGA3817 vector (Kim et al. 2009). The reporter (2 µg), effector (4 µg), and internal control (1 µg) plasmids were co-transfected into 5 × 10^4^ rice protoplasts using the PEG-mediated transfection method (Yoo et al. 2007). The transfected protoplasts were subsequently suspended in protoplast culture medium (0.4 mM mannitol, 4 mM MES, 15 mM MgCl_2_, pH 5.8), followed by overnight incubation in complete darkness for 12 h at room temperature. The LUC activity of each cell lysate was determined using the LUC reporter assay system kit (Promega).

### Statistical Analysis

Statistical analyses were performed using the two-tailed Student’s *t*-test with Microsoft Excel 2016. Significant differences between means are indicated by asterisks (*P < 0.05, **P < 0.01, and ***P < 0.001).

### Accession Numbers

The sequence data from this article can be found in the Rice Genome Annotation Project (http://rice.uga.edu/analyses_search_locus.shtml), GenBank (https://www.ncbi.nlm.nih.gov/genbank/), and EMBL’s European Bioinformatics Institute (EMBL-EBI; https://www.ebi.ac.uk/) databases under the accession numbers listed in Additional file 2: Table S1.

### Electronic Supplementary Material

Below is the link to the electronic supplementary material.

Supplementary Material 1

## Data Availability

All data supporting the findings of this study are available within the paper and within its supplementary materials published online.

## Abbreviations

ABA: Abscisic acid
ACC: 1-Aminocyclopropane-1-carboxylic acid
bHLH: basic Helix-Loop-Helix
BR: Brassinosteroid
CDG: Chlorophyll degradation gene
Chl: Chlorophyll
DAH: Days after heading
DDI: Day(s) of dark incubation
DIS: Dark-induced senescence
DT: Day(s) of treatment
DW: Dry weight
ELISA: Enzyme-linked immunosorbent assay
ET: Ethylene
FW: Fresh weight
JA: Jasmonic acid
LJ: Lamina joint
MeJA: Methyl jasmonate
NAC: NAM/ATAF1/2/CUC2
NS: Not significant
ORF: Open reading frame
PCR: Polymerase chain reaction
RT-qPCR: Reverse transcription quantitative real-time PCR
SA: Salicylic acid
SAG: Senescence-associated gene
Sen-TF: Senescence-associated transcription factor
TEM: Transmission electron microscopy
TF: Transcription factor
WT: Wild-type
