# Supplementary material for: Rice Basic Helix-Loop-Helix 079 (OsbHLH079) Delays Leaf Senescence by Attenuating ABA Signaling
Source: Rice (N Y). 2023 Dec 13;16:60. doi: 10.1186/s12284-023-00673-w (PMC10719235; doi:10.1186/s12284-023-00673-w)
Supplement: Supplementary file 1 — Supplementary Material 1 [file 12284_2023_673_MOESM1_ESM.docx]

**Additional file 1**


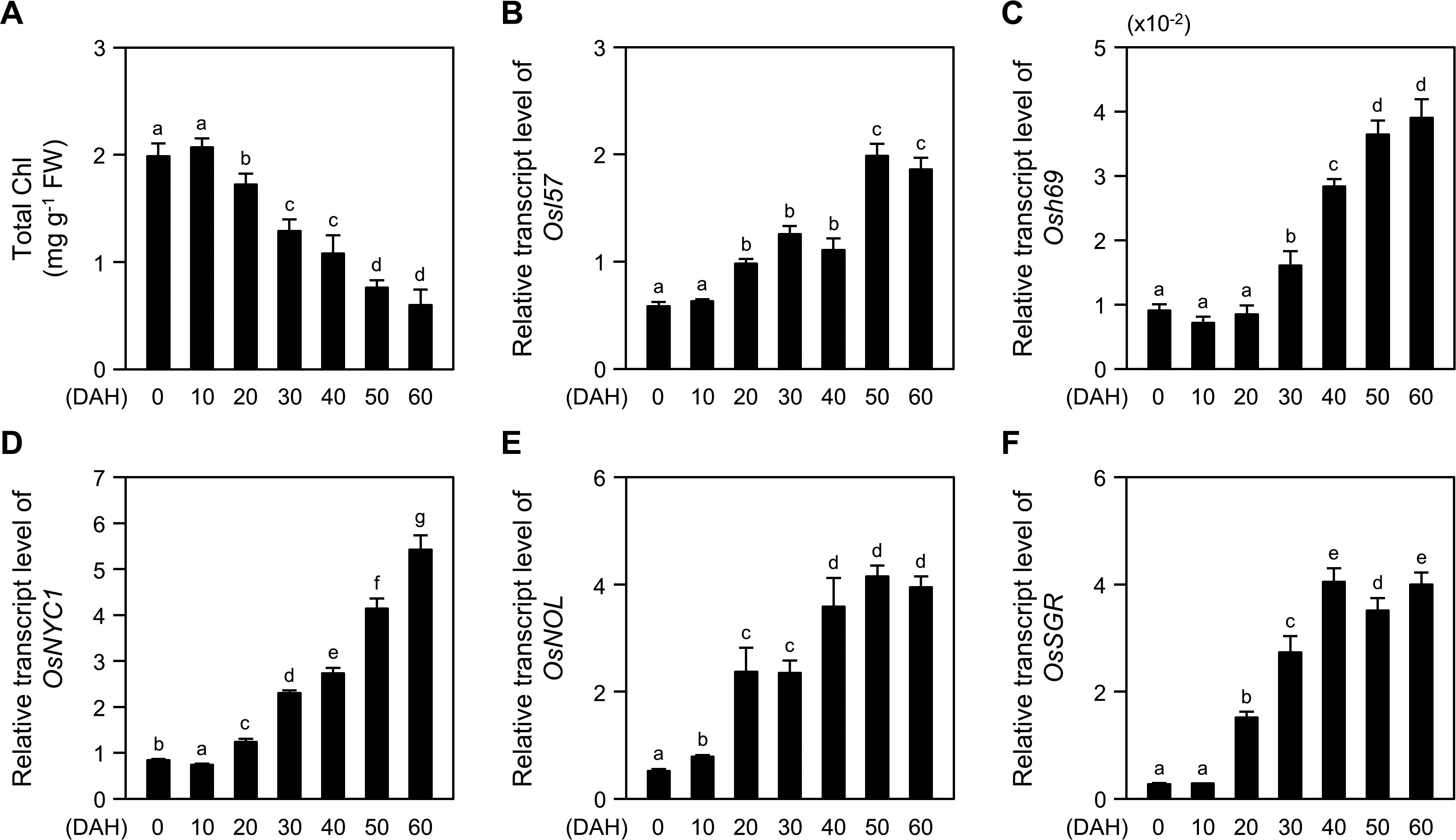


**Fig. S1** Changes in total chlorophyll content and expression levels of senescence-associated genes (SAGs) during natural senescence. **A** Total chlorophyll content in naturally senescing flag leaves of WT plants grown under natural long-day conditions in the paddy field. Flag leaves were collected at 10-day intervals after the heading stage. Means and standard deviations were calculated from data obtained from four independent plants. Significantly different values are indicated by distinct letters, as determined by one-way ANOVA and Duncan’s least significant range test (*P* < 0.05). Chl, chlorophyll; DAH, days after heading; FW, fresh weight. **B-F** Relative transcript levels of *Osl57* (**B**), *Osh69* (**C**), *OsNYC1* (**D**), *OsNOL* (**E**), and *OsSGR* (**F**) in flag leaves of WT plants. Total RNA samples from **Fig. 1A** were analyzed using RT-qPCR, with *GAPDH* serving as a reference for normalization. The presented values represent the means of four biological replicates, and the error bars indicate standard deviations. Statistically significant differences were determined through one-way ANOVA with Duncan’s least significant range test (*P* < 0.05). DAH, days after heading.


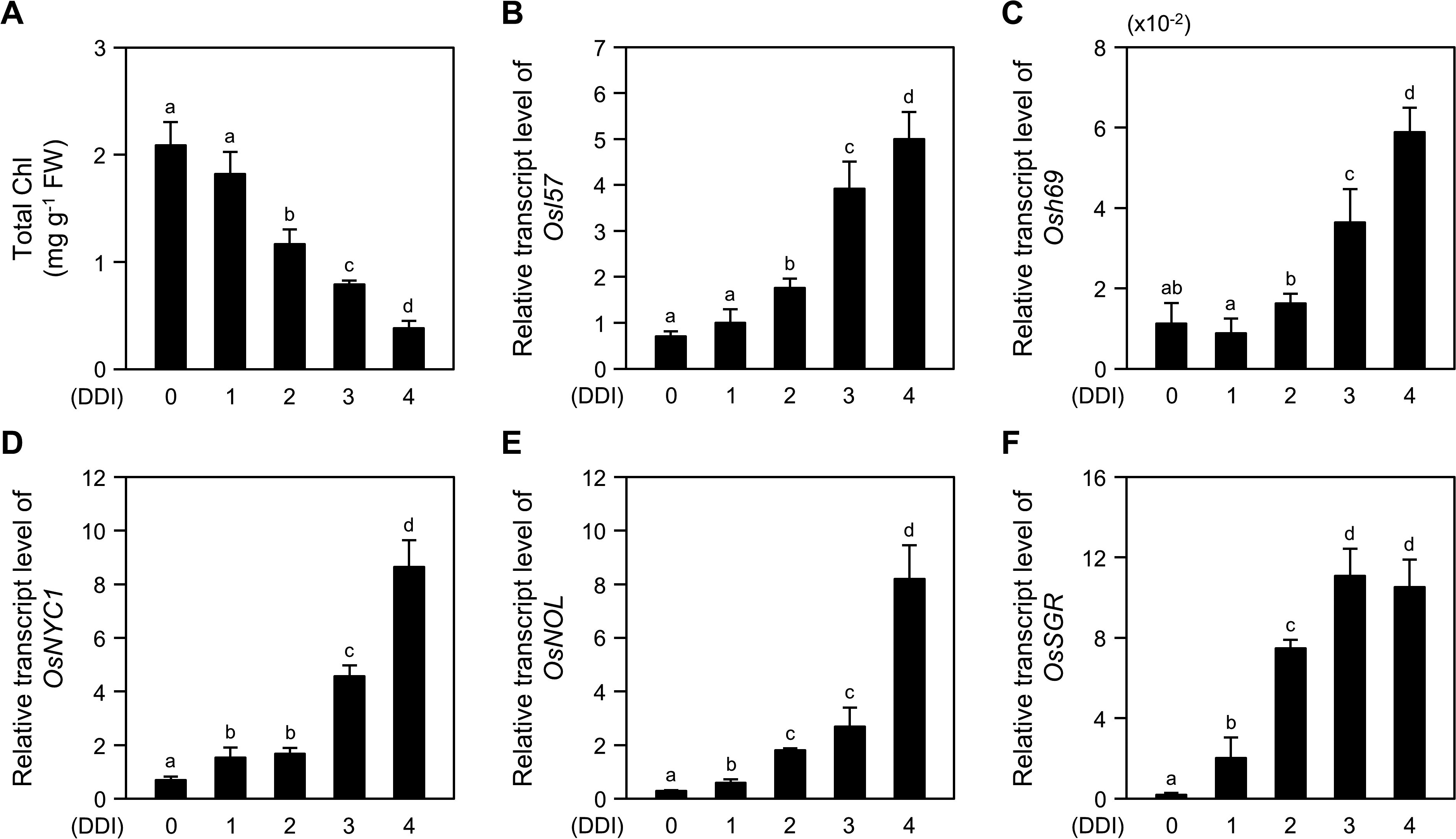


**Fig. S2** Alterations in total chlorophyll content and expression levels of senescence-associated genes (SAGs) during dark-induced senescence. **A** Total chlorophyll content in senescing flag leaves under dark conditions. Leaf discs were harvested from flag leaves of WT plants at the heading stage and incubated on a 3 mM MES buffer (pH 5.8) at 30°C in complete darkness until the specified DDI. The data were derived from four independent samples, each comprising approximately 10 mg of leaf discs. The values depicted in the graphs are averages, with error bars denoting standard deviations. Statistically different values were identified using one-way ANOVA with Duncan’s least significant range test (*P* < 0.05). Chl, chlorophyll; DDI, day(s) of dark incubation; FW, fresh weight. **B-F** Relative mRNA levels of *Osl57* (**B**), *Osh69* (**C**), *OsNYC1* (**D**), *OsNOL* (**E**), and *OsSGR* (**F**) in flag leaves of WT plants. Total RNA samples from **Fig. 1B** were subjected to RT-qPCR analysis, with *GAPDH* as a reference for normalization. The data represent the mean ± SD of four biological replicates (approximately 4 leaf discs per sample). Different letters indicate significantly different values, according to one-way ANOVA with Duncan’s least significant range test (*P* < 0.05). DDI, day(s) of dark incubation.


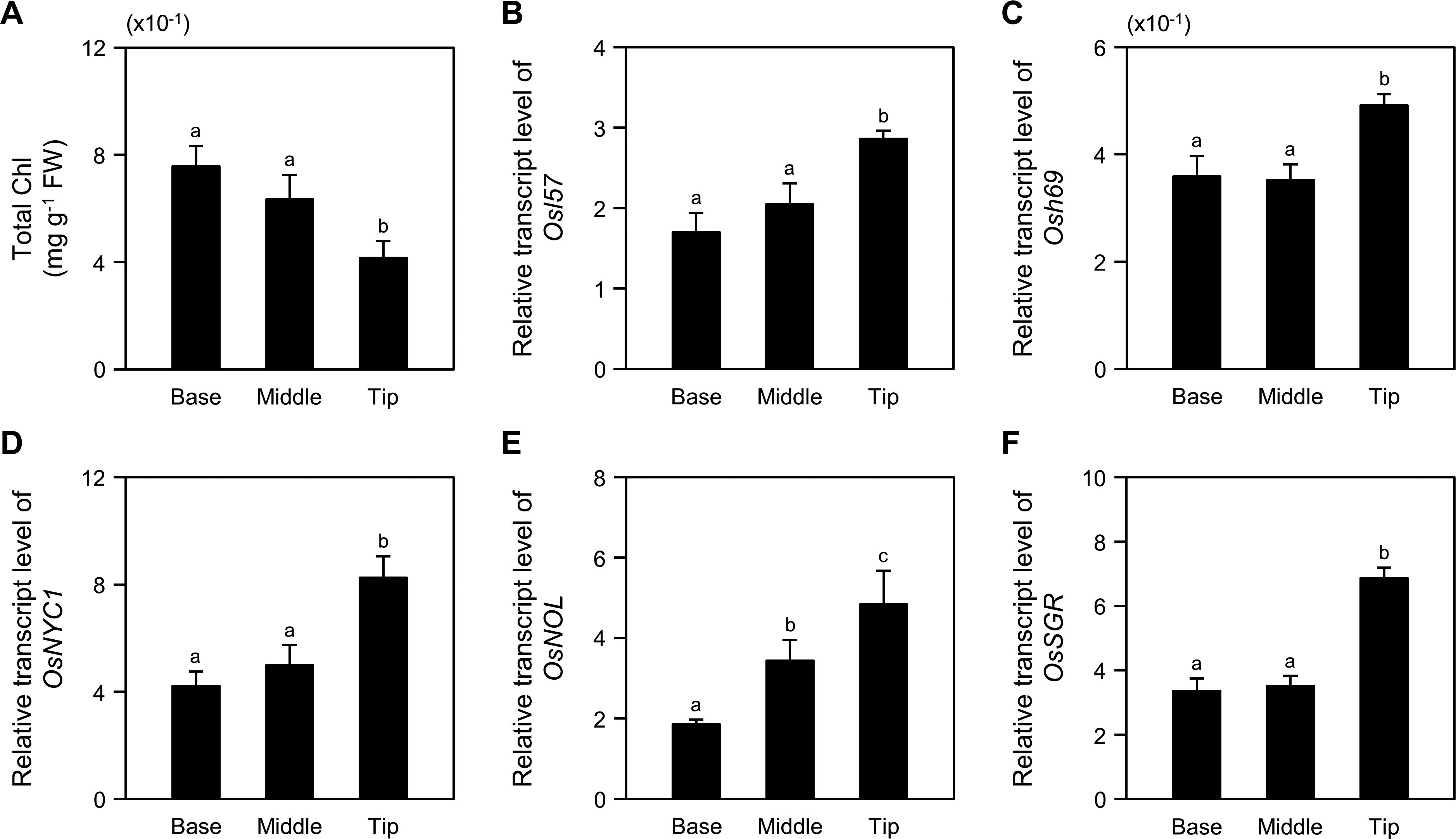


**Fig. S3** Total chlorophyll content and expression levels of senescence-associated genes (SAGs) in naturally senescing flag leaves. **A** Comparison of total chlorophyll content in different sectors of flag leaves. Each sector was sampled from WT plants at 50 DAH grown under natural day-night conditions in the paddy field. Averages and standard deviations were calculated from four biological replicates. Significantly different values are indicated by distinct letters, determined using one-way ANOVA with Duncan’s least significant range test (*P* < 0.05). Chl, chlorophyll; DAH, days after heading; FW, fresh weight. **B-F** Relative expression levels of *Osl57* (**B**), *Osh69* (**C**), *OsNYC1* (**D**), *OsNOL* (**E**), and *OsSGR* (**F**) in different sectors of naturally senescing flag leaves at 50 DAH. Total RNA samples from **Fig. 1C** were subjected to RT-qPCR analysis using *GAPDH* as an internal control. Data are presented as mean ± SD (*n* = 4). Different letters indicate significantly different values, determined through one-way ANOVA followed by Duncan’s least significant range test (*P* < 0.05).


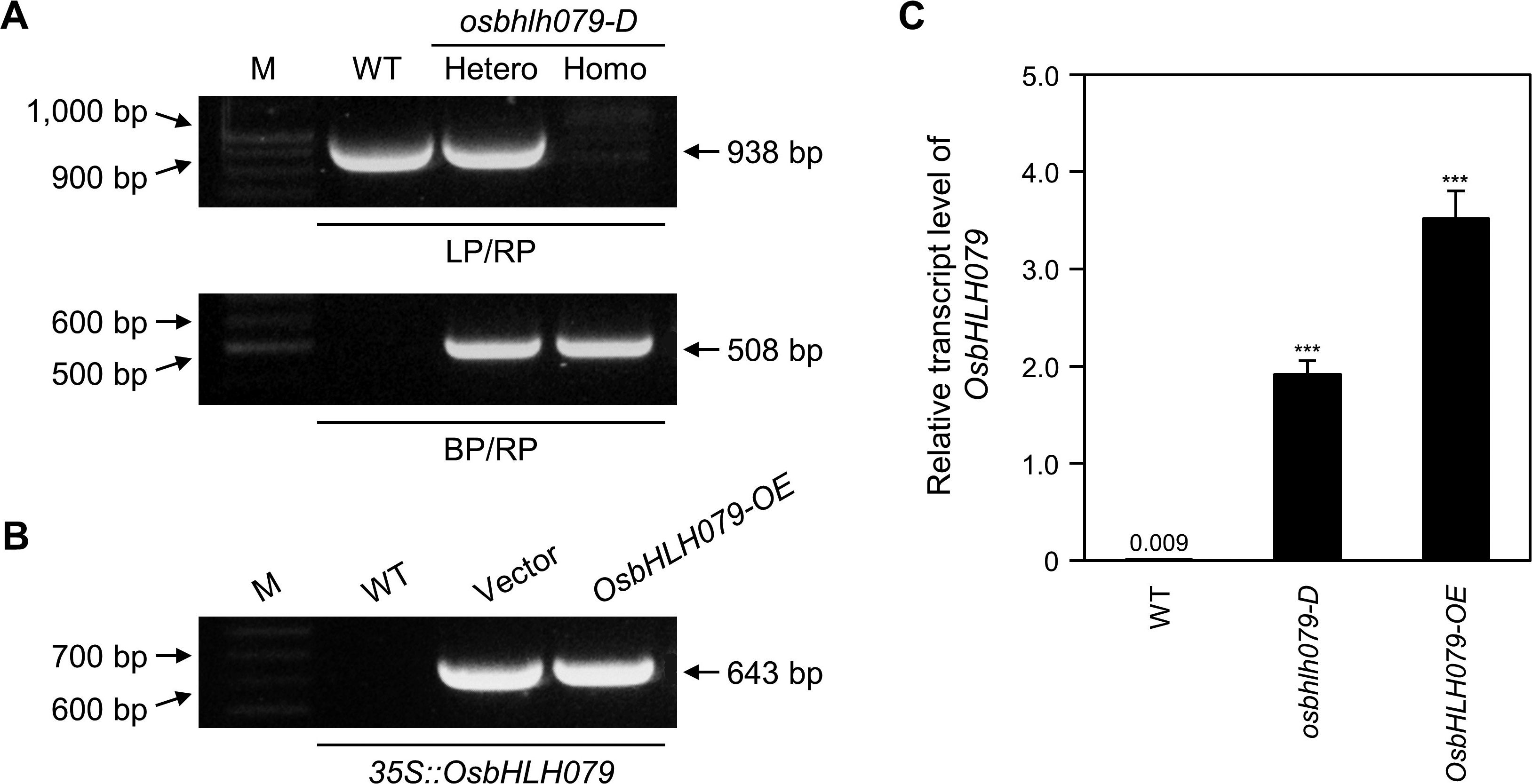


**Fig. S4** Details of *OsbHLH079* overexpression lines. **A** Detection of T-DNA insertion in *osbhlh079-D*. Genomic DNAs were extracted from the T_1_ segregating population and subjected to PCR analysis. The combination of LP and RP primers amplified a 938-bp fragment of the allele without T-DNA, while the combination of BP and RP primers amplified a 508-bp fragment of the allele with T-DNA. T_1_ plants with homozygous T-DNA insertion were selected and used for further study. BP, border primer; LP, left primer; M, marker; RP, right primer. **B** Genomic PCR analysis confirming rice transformation in *OsbHLH079-OE*. The *35S::OsbHLH079* construct was introduced into rice callus to generate *OsbHLH079-OE*. Genomic DNA was isolated from the resulting T_0_ plants and analyzed using a primer set amplifying part of the *35S::OsbHLH079* construct. WT and the vector containing the *35S::OsbHLH079* construct were used as negative and positive controls, respectively. M, marker. **C** Relative expression levels of *OsbHLH079* in WT, *osbhlh079-D*, and *OsbHLH079-OE*. Total RNAs were isolated from flag leaves at the heading stage, grown under natural long-day conditions in the paddy field, and subjected to RT-qPCR analysis, using *GAPDH* used as a reference for normalization. The data are presented as mean ± SD (*n* = 4). Asterisks indicate significant differences compared to WT, as determined by the two-tailed Student’s *t*-test (****P* < 0.001).


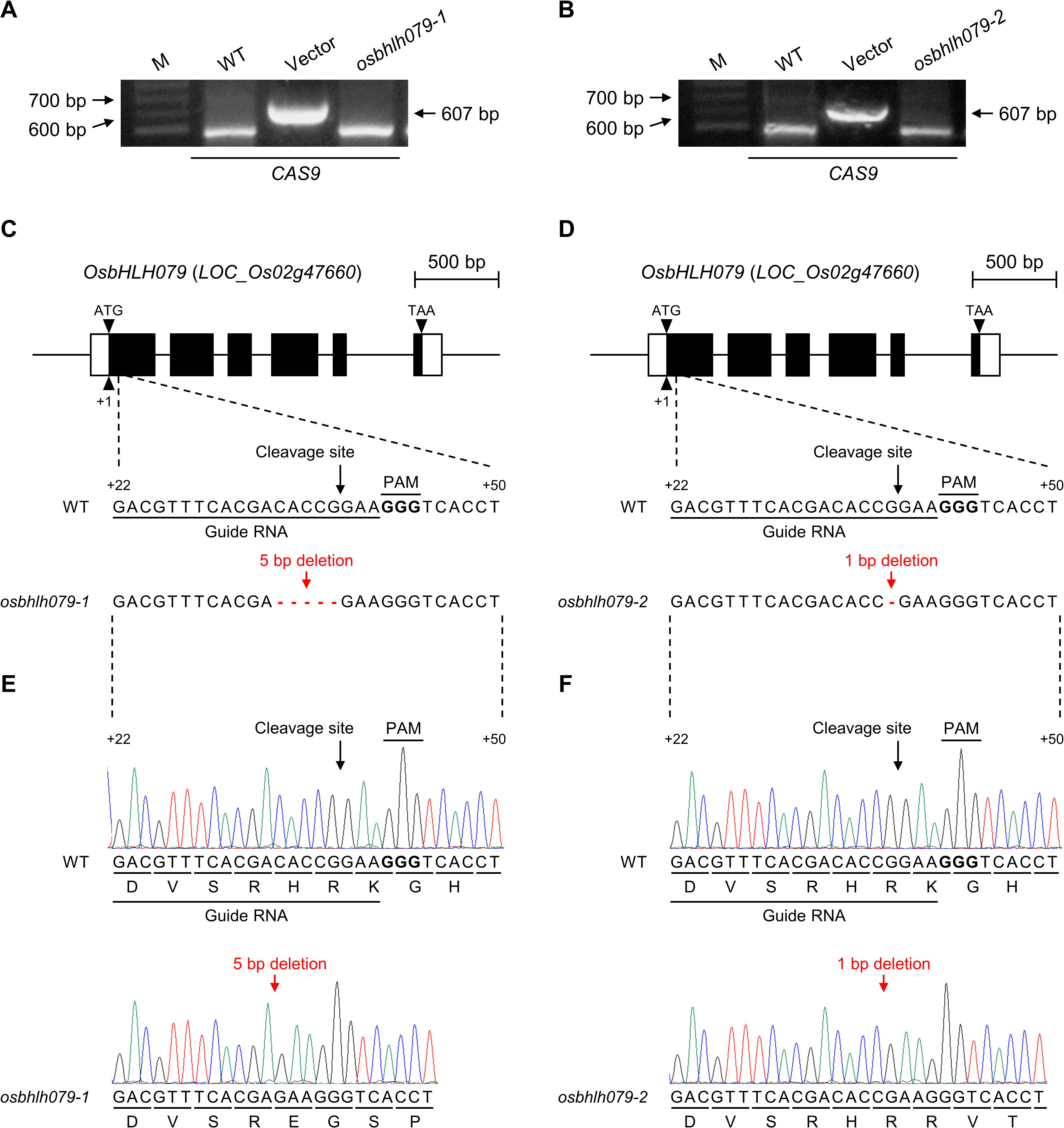


**Fig. S5** Description of *osbhlh079* knockout mutants. **A, B** PCR verification of the T-DNA-free *osbhlh079-1* (**A**) and *osbhlh079-2* (**B**) mutants. T-DNA, encoding both the *OsbHLH079*-targeted guide RNA and the Cas9 protein, was introduced into rice callus to generate *osbhlh079-1* and *osbhlh079-2* mutants. Genomic DNAs were extracted from the resulting T_1_ segregating population and subjected to PCR analysis using a primer set that amplifies a 607-bp fragment of the *Cas9* gene, aiming to obtain T-DNA-free *osbhlh079-1* and *osbhlh079-2* mutants for further study. WT and the vector harboring the *Cas9* gene were used as the negative and positive controls, respectively. M, marker. **C, D** Schematic representation of the target site for the *OsbHLH079*-specific guide RNA. The PAM is highlighted in bold, and the 20-nt spacer region is underlined. The black arrow indicates the location of the Cas9 cleavage site, which is 3-4 bp upstream of the PAM. The positions of the 5-bp deletion in *osbhlh079-1* (**C**) and the 1-bp deletion in *osbhlh079-2* (**D**) are highlighted in red. Nucleotide numbering is relative to the ATG start codon. PAM, protospacer adjacent motif. **E, F** Chromatograms of direct sequencing of genomic PCR products in the *osbhlh079-1* (**E**) and *osbhlh079-2* (**F**) mutants. The *OsbHLH079* genomic DNA regions surrounding the target site described in (**C**, **D**) were amplified, and the resulting PCR products were subjected to direct sequencing. The sequencing chromatograms of *osbhlh079-1* (**E**) and *osbhlh079-2* (**F**) were aligned with those of WT.


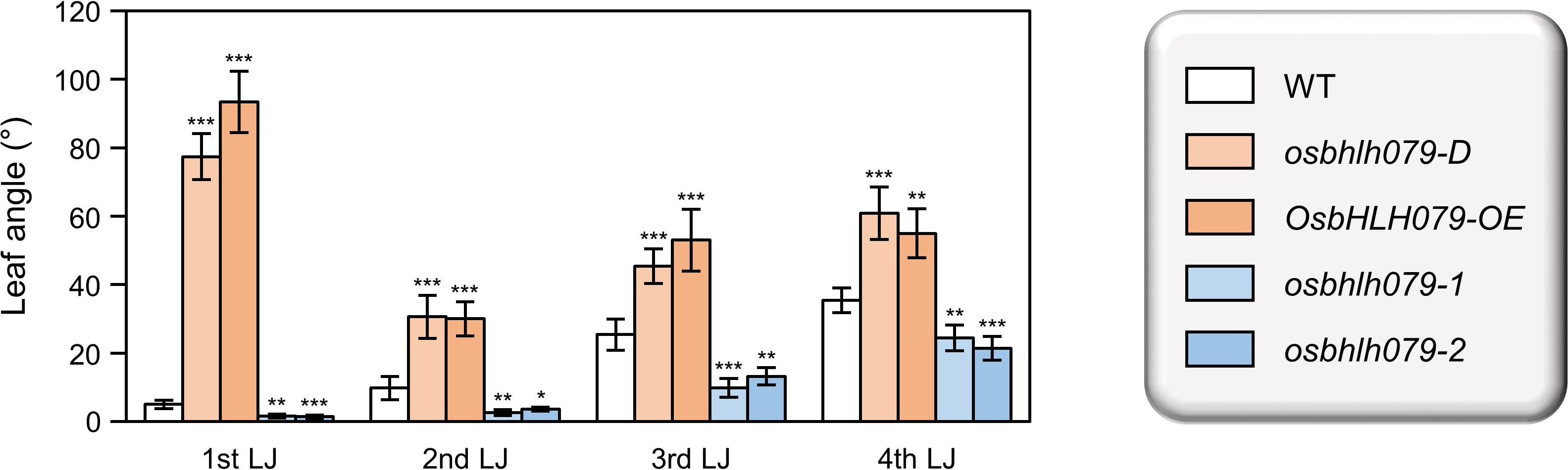


**Fig. S6** Leaf angle phenotypes of *osbhlh079-D*, *OsbHLH079-OE*, *osbhlh079-1*, and *osbhlh079-2*. Rice plants were grown under natural long day conditions in the field. At the heading stage, the leaf blade angles were measured in relation to the axis of the leaf sheath using a protractor. Means and standard deviations were calculated based on measurements from five independent plants, with five leaf angles recorded per plant. The two-tailed Student’s *t*-test was used to assess significant differences between means (**P* < 0.05, ***P* < 0.01, and ****P* < 0.001). LJ, lamina joint.


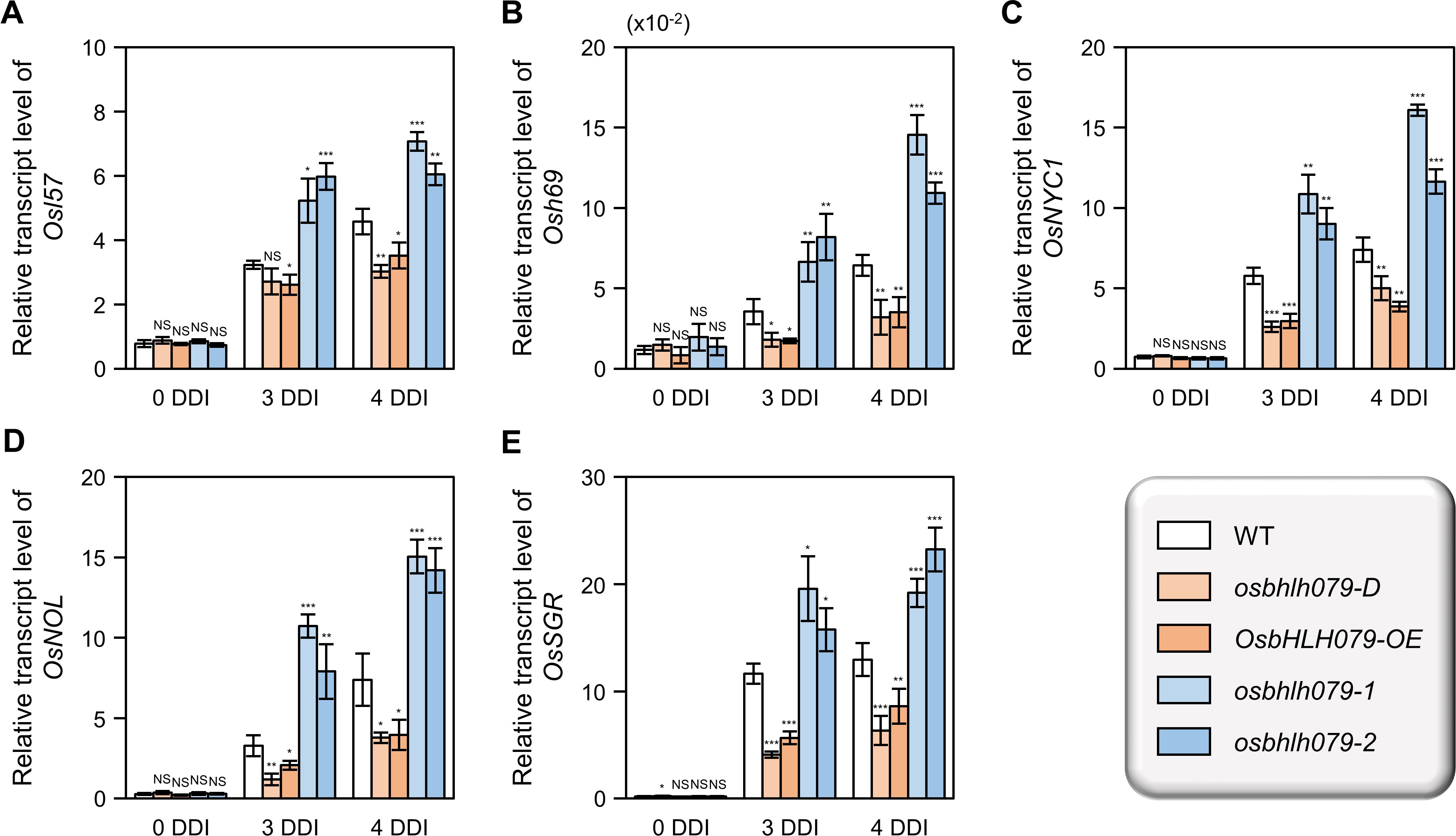


**Fig. S7** Expression profiles of senescence-associated genes and chlorophyll degradation genes in flag leaves during dark-induced senescence. **A-E** Relative transcript levels of *Osl57* (**A**), *Osh69* (**B**), *OsNYC1* (**C**), *OsNOL* (**D**), and *OsSGR* (**E**) in WT, *osbhlh079-D*, *OsbHLH079-OE*, *osbhlh079-1*, and *osbhlh079-2*. Flag leaf discs were collected at the heading stage from rice plants grown under natural field conditions. The leaf discs were then floated on a 3 mM MES buffer (pH 5.8) for specified time periods at 30°C in complete darkness and used for RT-qPCR analysis. The expression levels of each gene were normalized to that of *GAPDH*, serving as an internal control. The presented values represent the means of four biological replicates, with each replicate consisting of five leaf discs, and the error bars indicate the standard deviations. Asterisks on the bars denote statistically significant differences compared to WT transcript levels (Student’s *t*-test; **P* < 0.05, ***P* < 0.01, and ****P* < 0.001). DDI, day(s) of dark incubation; NS, not significant.


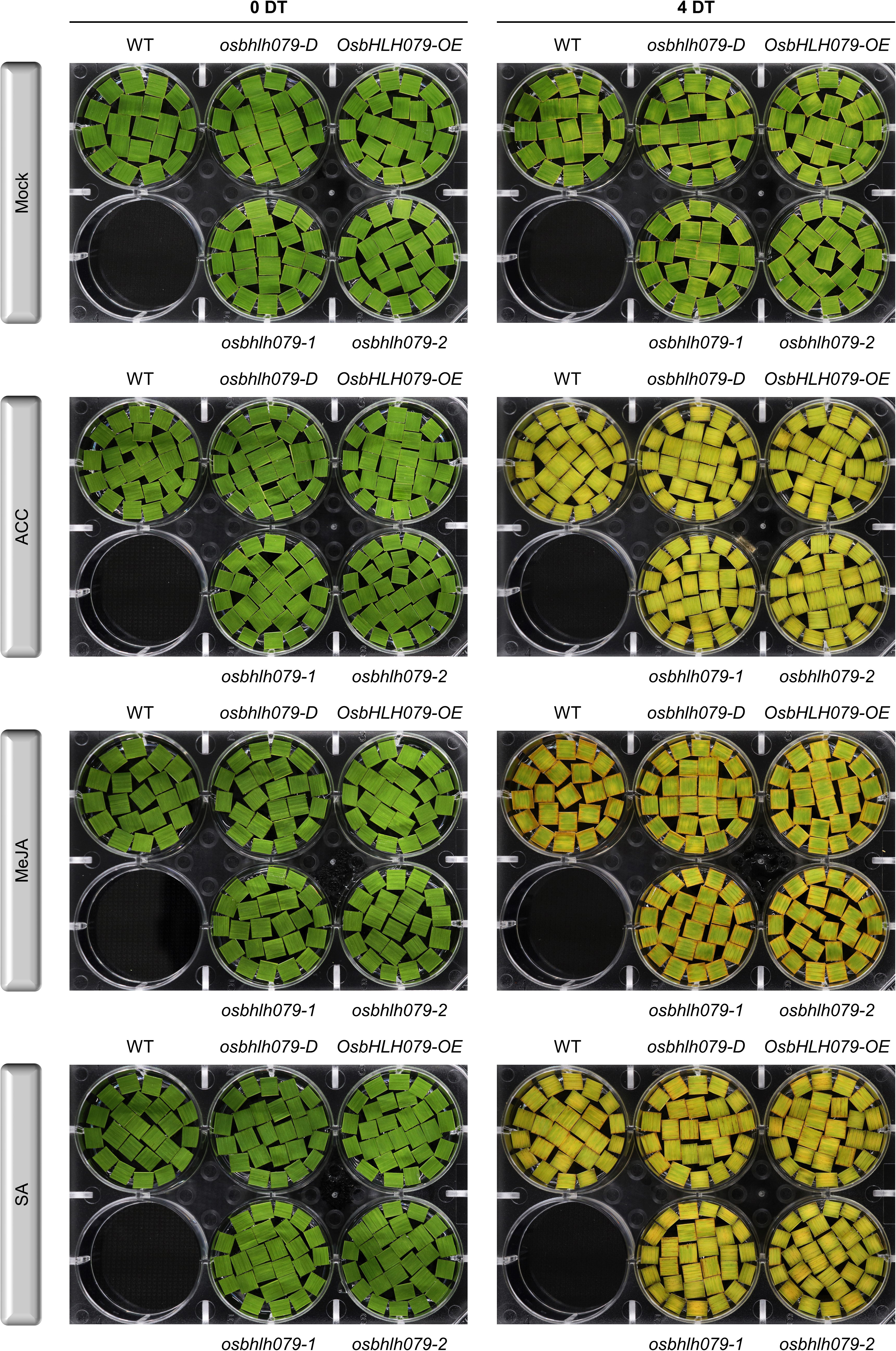


**Fig. S8** Leaf senescence phenotypes of *osbhlh079-D*, *OsbHLH079-OE*, *osbhlh079-1*, and *osbhlh079-2* in response to senescence-promoting phytohormone treatments. Flag leaves at the heading stage were collected from the plants grown under natural long-day conditions in the field. The leaves were then incubated on a 3 mM MES buffer (pH 5.8) supplemented with 20 mM ACC, 50 μM MeJA, or 100 μM SA. The incubation period was 4 days under constant light conditions at 30°C, abaxial side up. Leaf discs floating on a 3 mM MES buffer (pH 5.8) without any phytohormones served as the mock control. ACC, 1-aminocyclopropane-1-carboxylic acid; DT, day(s) of treatment; MeJA, methyl jasmonate; SA, salicylic acid.


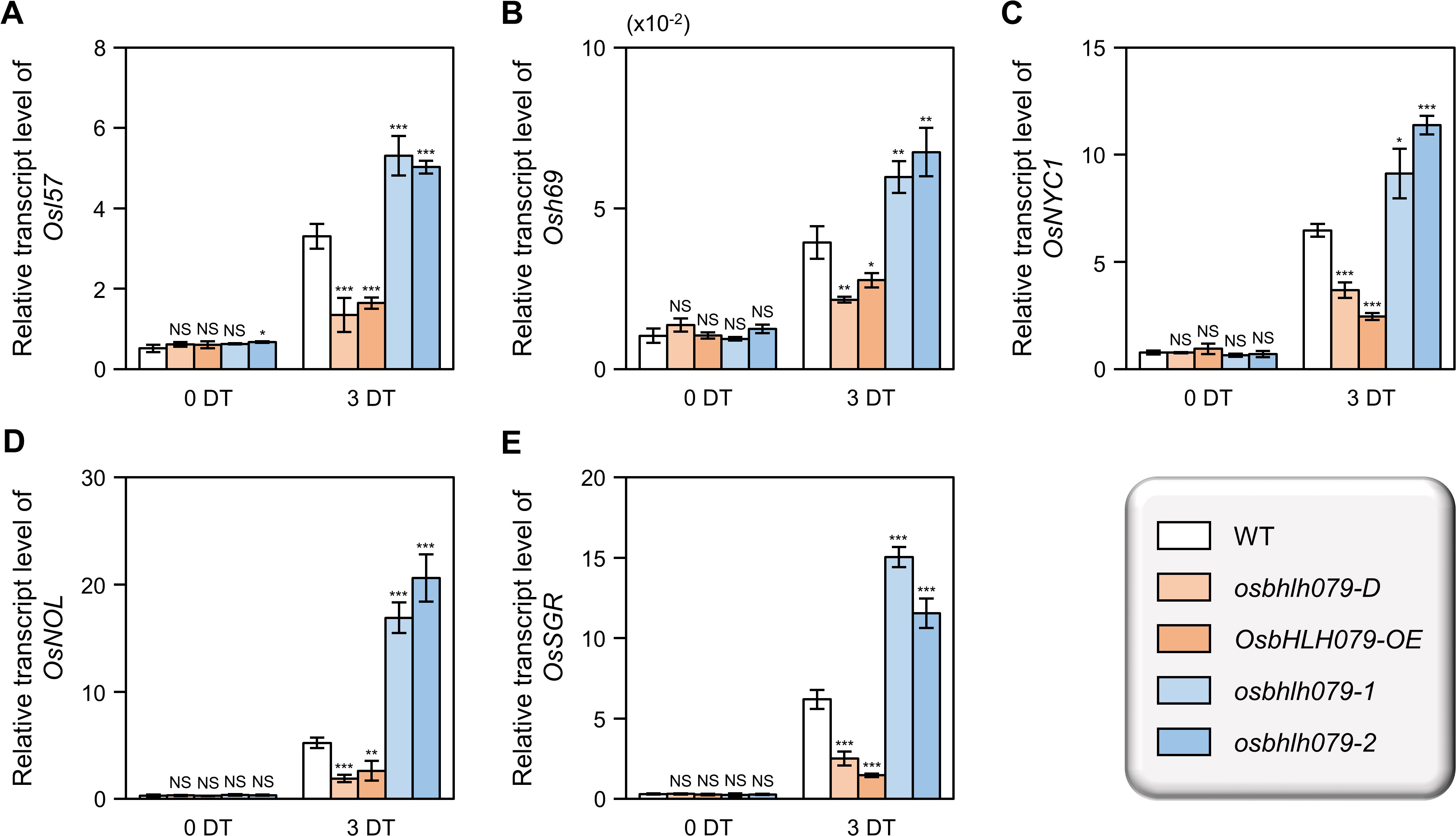


**Fig. S9** Expression analysis of senescence-associated genes and chlorophyll degradation genes in flag leaves during ABA-induced senescence. **A-E** Relative mRNA levels of *Osl57* (**A**), *Osh69* (**B**), *OsNYC1* (**C**), *OsNOL* (**D**), and *OsSGR* (**E**) in WT, *osbhlh079-D*, *OsbHLH079-OE*, *osbhlh079-1*, and *osbhlh079-2*. Flag leaf discs were sampled at the heading stage from rice plants grown under natural long-day conditions in the paddy field. The leaf discs were then treated with a 3 mM MES buffer (pH 5.8) containing 50 μM ABA for indicated time periods under continuous light conditions at 30°C and subjected to RT-qPCR analysis. The mRNA levels of *GAPDH*, serving as a reference gene, were used for normalization. The values shown in the graphs are averages of four biological samples (around 5 leaf discs per sample), with error bars indicating standard deviations. Differences between the means were statistically analyzed using a two-tailed Student’s *t*-test (**P* < 0.05, ***P* < 0.01, and ****P* < 0.001). DT, day(s) of treatment; NS, not significant.


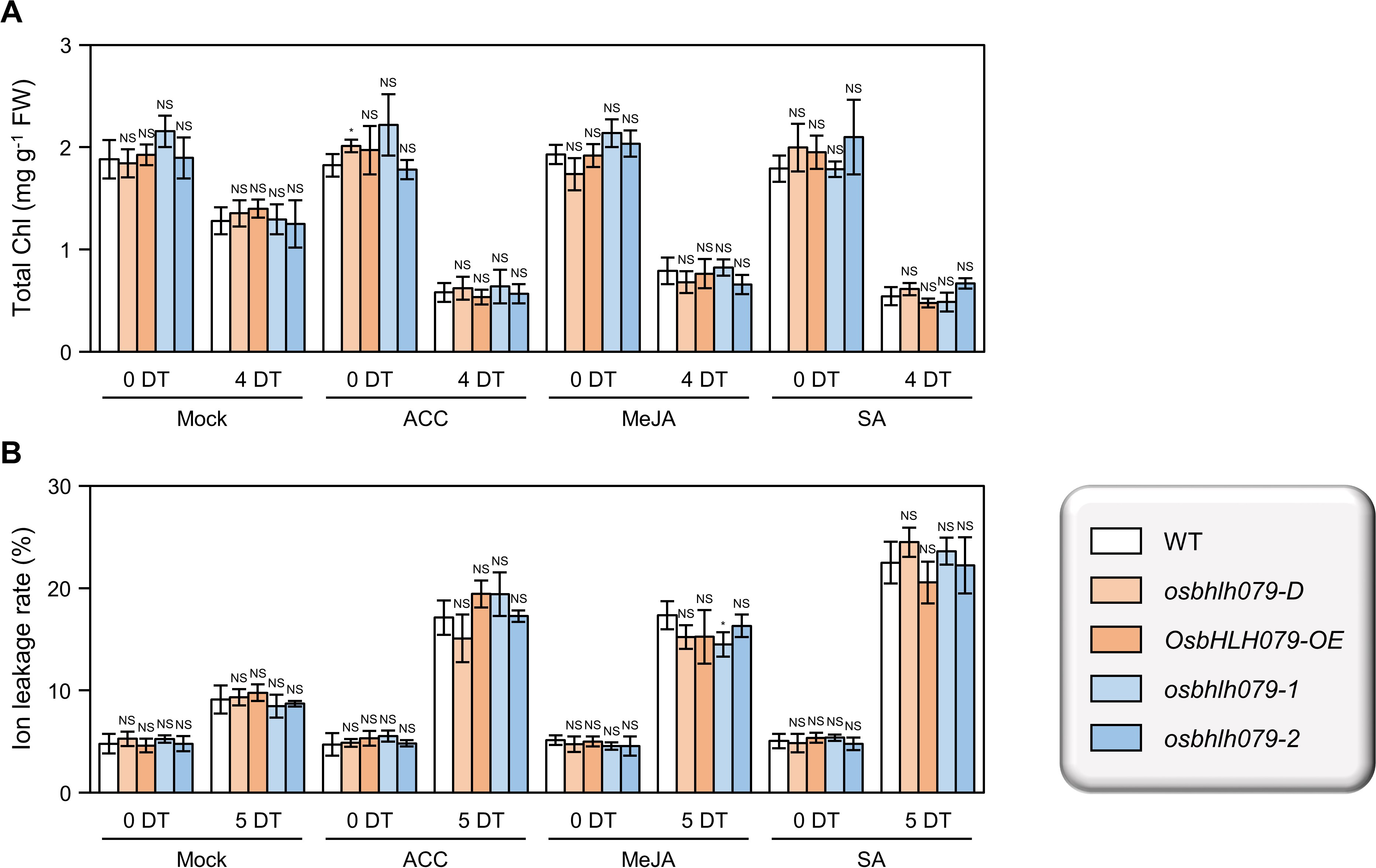


**Fig. S10** Total chlorophyll concentrations and ion leakage rates of the leaves of *osbhlh079-D*, *OsbHLH079-OE*, *osbhlh079-1*, and *osbhlh079-2* under phytohormone-mediated senescence conditions. Leaf discs shown in **Fig. S8** were collected at the indicated DT to measure the total chlorophyll content (**A**) and ion leakage rate (**B**). The data presented in (**A**, **B**) represent the mean ± SD of four replicates. Approximately 10 mg of leaf discs per replicate were used in (**A**) and five leaf discs per replicate were used in (**B**). Statistical analysis was conducted using the two-tailed Student’s *t*-test (**P* < 0.05). ACC, 1-aminocyclopropane-1-carboxylic acid; Chl, chlorophyll; DT, day(s) of treatment; FW, fresh weight; MeJA, methyl jasmonate; NS, not significant; SA, salicylic acid.


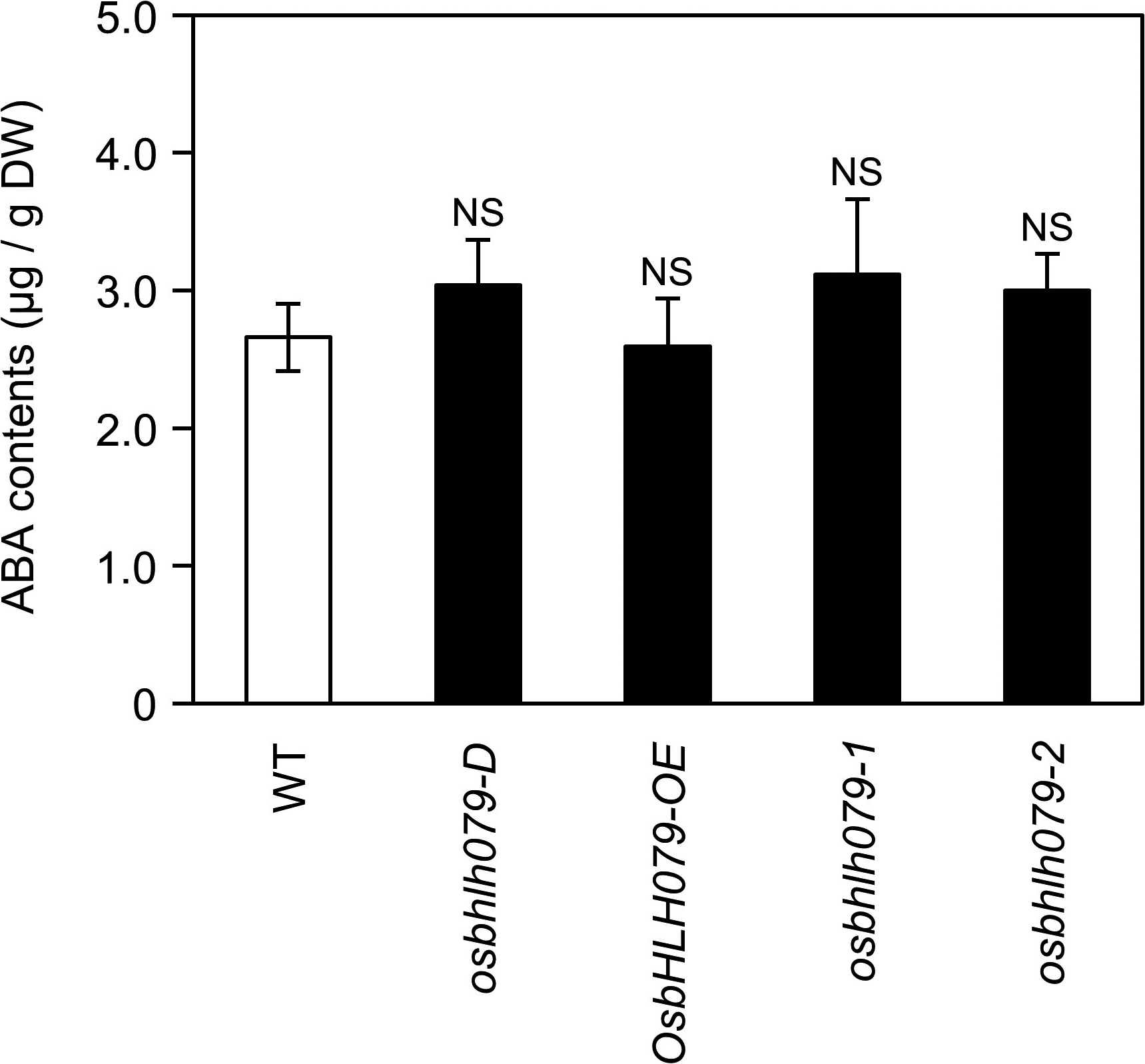


**Fig. S11** No difference in ABA levels among the flag leaves of WT, *osbhlh079-D*, *OsbHLH079-OE*, *osbhlh079-1*, and *osbhlh079-2*. Flag leaves were collected from plants at the heading stage for ABA quantification. Data represents the means and standard deviations derived from four biological replicates, with each replicate consisting of 10 flag leaves. Statistical analysis using a two-tailed Student’s *t*-test indicated no significant differences between means at the 0.05 significance level. This experiment was replicated twice with similar results. ABA, abscisic acid; DW, dry weight; NS. not significant.

**Additional file 2** - Kim et al.

**Table S1.** Genes described in this study and their corresponding locus IDs.

| **A. *OsbHLH079* and *GAPDH*** | | | | |  |
| --- | --- | --- | --- | --- | --- |
| **Gene** | **MSU_Locus** | **RAP_Locus** | **Accession No.** | **Reference** |  |
| *OsbHLH079* | LOC_Os02g47660 | Os02g0705500 | AK119183 | Seo et al. 2020 |  |
| *GAPDH* | LOC_Os04g40950 | Os04g0486600 | AK064960 | Jain et al. 2006 |  |
| **B. Senescence-associated genes** | | | | |  |
| **Gene** | **MSU_Locus** | **RAP_Locus** | **Accession No.** | **Reference** |  |
| *Osl2* | LOC_Os04g52450 | Os04g0614600 | AK102306 | Lee et al. 2001 |  |
| *Osl20* | LOC_Os12g08260 | Os12g0183100 | AK064862 |  |  |
| *Osl55* | LOC_Os12g41250 | Os12g0605800 | AK061906 |  |  |
| *Osl57* | LOC_Os02g57260 | Os02g0817700 | AK069768 |  |  |
| *Osl85* | LOC_Os07g34520 | Os07g0529000 | AK103744 |  |  |
| *Osh69* | LOC_Os08g38710 | Os08g0495800 | AK067229 |  |  |
| *OsSAG12-2* | LOC_Os08g44270 | Os08g0556900 | AK105612 | Singh et al. 2016 |  |
| **C. Chlorophyll degradation genes** | | | | |  |
| **Gene** | **MSU_Locus** | **RAP_Locus** | **Accession No.** | **Reference** |  |
| *OsNYC1* | LOC_Os01g12710 | Os01g0227100 | AK061638 | Kusaba et al. 2007 |  |
| *OsNOL* | LOC_Os03g45194 | Os03g0654600 | CB669634 | Sato et al. 2009 |  |
| *OsHCAR* | LOC_Os04g25400 | Os04g0320100 | AK068015 | Piao et al. 2017 |  |
| *OsSGR* | LOC_Os09g36200 | Os09g0532000 | AK105810 | Park et al. 2007 |  |
| *OsNYC3* | LOC_Os06g24730 | Os06g0354700 | AK066777 | Morita et al. 2009 |  |
| *OsPAO* | LOC_Os03g05310 | Os03g0146400 | AK120554 | Tang et al. 2011 |  |
| *OsRCCR1* | LOC_Os10g25030 | Os10g0389200 | CI479132 |  |  |
| **D. ABA signaling genes** | | | | |  |
| **Gene** | **MSU_Locus** | **RAP_Locus** | **Accession No.** | **Reference** |  |
| *OsABF2* | LOC_Os06g10880 | Os06g0211200 | AK105312 | Hossain et al. 2010a |  |
| *OsABF4* | LOC_Os09g28310 | Os09g0456200 | AK065873 | Lu et al. 2009 |  |
| *OsABI5* | LOC_Os01g64730 | Os01g0867300 | AK067919 | Hossain et al. 2010b |  |
| *OsbZIP23* | LOC_Os02g52780 | Os02g0766700 | AK072062 | Xiang et al. 2008 |  |
| *OsEEL* | LOC_Os07g48660 | Os07g0686100 | AK110915 | Yang et al. 2019 |  |
| *OsNAP* | LOC_Os03g21060 | Os03g0327800 | CI362055 | Liang et al. 2014 |  |
| **E. References** | | | | | |
| Hossain MA, Cho JI, Han M, Ahn CH, Jeon JS, An G, Park PB (2010a) The ABRE-binding bZIP transcription factor OsABF2 is a positive regulator of abiotic stress and ABA signaling in rice. J Plant Physiol 167(17):1512-1520. https://doi.org/10.1016/j.jplph.2010.05.008  Hossain MA, Lee Y, Cho JI, Ahn CH, Lee SK, Jeon JS, Kang H, Lee CH, An G, Park PB (2010b) The bZIP transcription factor OsABF1 is an ABA responsive element binding factor that enhances abiotic stress signaling in rice. Plant Mol Biol 72:557-566. https://doi.org/10.1007/s11103-009-9592-9  Jain M, Nijhawan A, Tyagi AK, Khurana JP (2006) Validation of housekeeping genes as internal control for studying gene expression in rice by quantitative real-time PCR. Biochem Biophys Res Commun 345(2):646-651. https://doi.org/10.1016/j.bbrc.2006.04.140  Kusaba M, Ito H, Morita R, Iida S, Sato Y, Fujimoto M, Kawasaki S, Tanaka R, Hirochika H, Nishimura M, Tanaka A (2007) Rice NON-YELLOW COLORING1 is involved in light-harvesting complex II and grana degradation during leaf senescence. Plant Cell 19(4):1362-1375. https://doi.org/10.1105/tpc.106.042911  Lee RH, Wang CH, Huang LT, Chen SCG (2001) Leaf senescence in rice plants: cloning and characterization of senescence up-regulated genes. J Exp Bot 52(358):1117-1121. https://doi.org/10.1093/jexbot/52.358.1117  Liang C, Wang Y, Zhu Y, Tang J, Hu B, Liu L, Ou S, Wu H, Sun X, Chu J, Chu C (2014) OsNAP connects abscisic acid and leaf senescence by fine-tuning abscisic acid biosynthesis and directly targeting senescence-associated genes in rice. Proc Natl Acad Sci USA 111(27):10013-10018. https://doi.org/10.1073/pnas.1321568111  Lu G, Gao C, Zheng X, Han B (2009) Identification of OsbZIP72 as a positive regulator of ABA response and drought tolerance in rice. Planta 229:605-615. https://doi.org/10.1007/s00425-008-0857-3  Morita R, Sato Y, Masuda Y, Nishimura M, Kusaba M (2009) Defect in non-yellow coloring 3, an α/β hydrolase-fold family protein, causes a stay-green phenotype during leaf senescence in rice. Plant J 59(6):940-952. https://doi.org/10.1111/j.1365-313X.2009.03919.x  Park SY, Yu JW, Park JS, Li J, Yoo SC, Lee NY, Lee SK, Jeong SW, Seo HS, Koh HJ, Jeon JS, Park YI, Paek NC (2007) The senescence-induced staygreen protein regulates chlorophyll degradation. Plant Cell 19(5):1649-1664. https://doi.org/10.1105/tpc.106.044891  Piao W, Han SH, Sakuraba Y, Paek NC (2017) Rice 7-hydroxymethyl chlorophyll *a* reductase is involved in the promotion of chlorophyll degradation and modulates cell death signaling. Mol Cells 40(10):773-786. https://doi.org/10.14348/molcells.2017.0127  Sato Y, Morita R, Katsuma S, Nishimura M, Tanaka A, Kusaba M (2009) Two short-chain dehydrogenase/reductases, NON-YELLOW COLORING 1 and NYC1-LIKE, are required for chlorophyll *b* and light-harvesting complex II degradation during senescence in rice. Plant J 57(1):120-131. https://doi.org/10.1111/j.1365-313X.2008.03670.x  Seo H, Kim SH, Lee BD, Lim JH, Lee SJ, An G, Paek NC (2020) The rice *basic Helix-Loop-Helix 79* (*OsbHLH079*) determines leaf angle and grain shape. Int J Mol Sci 21(6):2090. https://doi.org/10.3390/ijms21062090  Singh S, Singh A, Nandi AK (2016) The rice *OsSAG12-2* gene codes for a functional protease that negatively regulates stress-induced cell death. J Biosci 41(3):445-453. https://doi.org/10.1007/s12038-016-9626-9  Tang Y, Li M, Chen Y, Wu P, Wu G, Jiang H (2011) Knockdown of *OsPAO* and *OsRCCR1* cause different plant death phenotypes in rice. J Plant Physiol 168(16):1952-1959. https://doi.org/10.1016/j.jplph.2011.05.026  Xiang Y, Tang N, Du H, Ye H, Xiong L (2008) Characterization of OsbZIP23 as a key player of the basic leucine zipper transcription factor family for conferring abscisic acid sensitivity and salinity and drought tolerance in rice. Plant Physiol 148(4):1938-1952. https://doi.org/10.1104/pp.108.128199  Yang S, Xu K, Chen S, Li T, Xia H, Chen L, Liu H, Luo L (2019) A stress-responsive bZIP transcription factor *OsbZIP62* improves drought and oxidative tolerance in rice. BMC Plant Biol 19:260. https://doi.org/10.1186/s12870-019-1872-1 | | | | | |

**Table S2.** Primers used in this study.

| **A. Cloning** | | |
| --- | --- | --- |
| **Primer name** | **Forward primer (5' to 3')** | **Reverse primer (5' to 3')** |
| *OsbHLH079* coding sequence | ATGGCGCAATGCGGCGGCGGCGAC | TTACATTTCCATTTTGAGATTGTC |
| *osbhlh079* guide RNA | GGCAGACGTTTCACGACACCGGAA | AAACTTCCGGTGTCGTGAAACGTC |
| **B. Genotyping** | | |
| **Primer name** | **Forward primer (5' to 3')** | **Reverse primer (5' to 3')** |
| *osbhlh079-D* LP/RP | CAGCAGATCCACACAAACGTTC | CTCATCAATTGGCCCTCCTGT |
| *osbhlh079-D* BP/RP | CTAGAGTCGAGAATTCAGTACA | CTCATCAATTGGCCCTCCTGT |
| *35S::OsbHLH079* | CTATCCTTCGCAAGACCCTT | TGGATGTAGTCGGTCTTCTGG |
| *CAS9* | CTCTCTAGGAAGCTCATCAACGGC | TGTTCTTGTCGGAGCGGGTG |
| **C. RT-qPCR** | | |
| **Primer name** | **Forward primer (5' to 3')** | **Reverse primer (5' to 3')** |
| *GAPDH* qPCR | AAGCCAGCATCCTATGATCAGATT | CGTAACCCAGAATACCCTTGAGTTT |
| *OsbHLH079* qPCR | AGATGAGCCCCTTGCAGCAGAT | GCCCGGTGGTGAGTACTGGTT |
| *Osl2* qPCR | CGCAGACAACAAATCGCCAA | CTCCAGCAACTCTAACCAGCA |
| *Osl20* qPCR | GCGGCACAAGTGAGGGAGAC | TTGGGGTGCTGATTGTCCAG |
| *Osl55* qPCR | ACCCAGGCAGAGATTCACGAC | ATTATTTGCTTGCTTTTGGACGGC |
| *Osl57* qPCR | ACCCTAAAGTAAATGAAGTC | CCTGCTCTTGTCTTGTTA |
| *Osl85* qPCR | AAGACCTTCTCTGACTGCGT | AGTCCCAGAACAGGGATGTG |
| *Osh69* qPCR | CCACAACACGGATAACTT | GGTGAACACTATGGAACA |
| *OsSAG12-2* qPCR | CCGTTTGGAGGCGATTGATC | GCATGCAATCAAATCAACTACAGG |
| *OsNYC1* qPCR | GAATCCGTAATTGGGCTGAA | CTGGAAGAGGTCCACCTGAG |
| *OsNOL* qPCR | TCTTATGTCTGGTGCCACAAC | GGATGTAGGTTGGTTTCATGG |
| *OsHCAR* qPCR | CTGAATCTGATTGGGCCGAAG | TAGGAAGGAATGTGCTGCTCC |
| *OsSGR* qPCR | TTCGTAGTGGCATTTAGCTCAGC | AGCTCACCACACTCATTCCCT |
| *OsNYC3* qPCR | TGTCGTTGCCATGTGAAGAT | TTGGTCACGCCACAAATCTA |
| *OsPAO* qPCR | GTGTTGCCTTCCACTGTCCT | ACTGAACATCCGCAGGAATC |
| *OsRCCR1* qPCR | CCAGTCCTCACTACACTGCAA | AGAAGGTCCAGAAGCACCAC |
| *OsABF2* qPCR | GCTGAGGTGGCAAAACTGAAG | GTGTTCGTCGGAGGCAAAAT |
| *OsABF4* qPCR | GGGGACCAAGAATCATACTGC | AGGAAGCAGGTACAGATATCAGAT |
| *OsABI5* qPCR | ACGCAACTCGAGGAGGAGAA | TGATGACTGGAACAACCATTTCC |
| *OsbZIP23* qPCR | CGCCAGAGGAAACAGGCATA | GGTCCAACTTGTCGGCTCAT |
| *OsEEL* qPCR | CGGCCTCTTTCTAATCAACCAACC | ATCCCCAAGTACAAACCCCCA |
| *OsNAP* qPCR | CAAGAAGCCGAACGGTTC | GTTAGAGTGGAGCAGCAT |
| **D. Dual-luciferase reporter assay** | | |
| **Primer name** | **Forward primer (5' to 3')** | **Reverse primer (5' to 3')** |
| *OsbHLH079* | AGTGTTAACAAGCTTATGGCGCAATGCGGC | GGGTGTACAACGCGTCATTTCCATTTTGAGATTGT |
| *proOsABF2* | GCCAGTGCCAAGCTTACACCTAGTTCTCGTATTATGCGT | TCTTCCATGGTCGACCTCCTCTCCCACCTCAAAATCC |
| *proOsABF4* | GCCAGTGCCAAGCTTCCTGTGGACCTTTTTGCCTGC | TCTTCCATGGTCGACTCAGCATCAAGCAACGCACCA |
| *proOsABI5* | GCCAGTGCCAAGCTTCCGTACAAACCTGCAACGTACT | TCTTCCATGGTCGACCGAGATCCGATGCCGTGTG |
| *proOsNAP* | GCCAGTGCCAAGCTTGGGACGCTCCAGTGTTTCTGT | TCTTCCATGGTCGACGCGGAGGTAGTGCACGATCA |

**Table S3.** Microwave settings for TEM sample preparation.

| **Step** | | **Time** | **Power** | ***T*_max_** |
| --- | --- | --- | --- | --- |
| Post-fixation | 1% osmium tetroxide | 2 min On, 2 min Off, 4 min On, 2 min Off,  4 min On, 2 min Off, and 4 min On | 100 W | 30 °C |
| *En bloc* staining | 0.5% uranyl acetate | 1 min On, 1 min Off, 1 min On, 1 min Off, and 1 min On | 100 W | 30 °C |
| Dehydration | 30% ethanol | 50 sec On | 150 W | 35 °C |
|  | 50% ethanol | 50 sec On | 150 W | 35 °C |
|  | 70% ethanol | 50 sec On | 150 W | 35 °C |
|  | 90% ethanol | 50 sec On | 150 W | 35 °C |
|  | 100% ethanol | 50 sec On | 150 W | 35 °C |
|  | 100% ethanol | 50 sec On | 150 W | 35 °C |
|  | 100% ethanol | 50 sec On | 150 W | 35 °C |
| Transition | 100% propylene oxide | 50 sec On | 150 W | 35 °C |
|  | 100% propylene oxide | 50 sec On | 150 W | 35 °C |
|  | 20% Spurr’s resin | 10 min On | 200 W | 40 °C |
|  | 40% Spurr’s resin | 10 min On | 200 W | 40 °C |
|  | 60% Spurr’s resin | 10 min On | 200 W | 40 °C |
|  | 80% Spurr’s resin | 10 min On | 200 W | 40 °C |
|  | 100% Spurr’s resin | 10 min On | 200 W | 40 °C |
|  | 100% Spurr’s resin | 10 min On | 200 W | 40 °C |
